# Optimizing Fixation Filters for Eye-Tracking on Small Screens

**DOI:** 10.3389/fnins.2021.578439

**Published:** 2021-11-08

**Authors:** Julia Trabulsi, Kian Norouzi, Seidi Suurmets, Mike Storm, Thomas Zoëga Ramsøy

**Affiliations:** ^1^Facebook Inc, New York, NY, United States; ^2^Neurons Inc., Department of Applied Neuroscience, Taastrup, Denmark; ^3^Faculty of Management, University of Tehran, Tehran, Iran; ^4^Department of Marketing, Copenhagen Business School, Copenhagen, Denmark; ^5^Faculty of Neuroscience, Singularity University, Santa Clara, CA, United States

**Keywords:** mobile eye-tracking, smartphone, mobile environment, social media marketing, validity, reliability, fixation algorithms

## Abstract

The study of consumer responses to advertising has recently expanded to include the use of eye-tracking to track the gaze of consumers. The calibration and validation of eye-gaze have typically been measured on large screens in static, controlled settings. However, little is known about how precise gaze localizations and eye fixations are on smaller screens, such as smartphones, and in moving feed-based conditions, such as those found on social media websites. We tested the precision of eye-tracking fixation detection algorithms relative to raw gaze mapping in natural scrolling conditions. Our results demonstrate that default fixation detection algorithms normally employed by hardware providers exhibit suboptimal performance on mobile phones. In this paper, we provide a detailed account of how different parameters in eye-tracking software can affect the validity and reliability of critical metrics, such as Percent Seen and Total Fixation Duration. We provide recommendations for producing improved eye-tracking metrics for content on small screens, such as smartphones, and vertically moving environments, such as a social media feed. The adjustments to the fixation detection algorithm we propose improves the accuracy of Percent Seen by 19% compared to a leading eye-tracking provider’s default fixation filter settings. The methodological approach provided in this paper could additionally serve as a framework for assessing the validity of applied neuroscience methods and metrics beyond mobile eye-tracking.

## Introduction

With the advent of smartphones around the turn of this decade, the use of mobile phones has now expanded well beyond regular phone calls. Smartphones are today becoming devices that are used ubiquitously around the globe (Wike and Oates, 2014), and are used not only for social interaction but for additional behaviors, such as the consumption of information and entertainment and the ordering of goods and services. As a result, there is increased interest in understanding the use of smartphones as part of the customer journey. Here, the combination of small screens packed with information at a close focal distance, coupled with vertical movement during scrolling behaviors, poses a particular challenge to those who want to use methods such as eye-tracking glasses with off-the-shelf fixation detection software in assessing different aspects of customer attention.

As interest and usage of eye-tracking methods have increased, so has the discussion of how to define and operationalize fixations, particularly how to define and measure fixations so that meaningful visual processing is captured under a specific environment. Indeed, much work has been performed related to the validity and reliability of stationary eye-tracking measures (Blignaut, 2009; Nyström et al., 2013), and it has become clear that there is no universal definition for a fixation, which varies operationally in different settings. For example, comparing the results of ten eye-movement event-detection algorithms with the ratings of two human experts, Andersson et al. (2017) showed that the resulting event durations varied substantially depending on what algorithm was used. The authors also found that while the existing approaches work well for static stimuli, the detection of fixations and saccades for dynamic stimuli is barely better than chance (Andersson et al., 2017). Additionally, Tobii, a leading eye-tracking vendor, explicitly states that the fixation filter settings have a substantial impact on the results of eye-tracking research (Olsen, 2012). With more recent advances that allow eye-tracking in mobile environments, this discussion about the validity and reliability of eye-tracking measures has received renewed interest, and along with it, a desire to establish an operationalized definition of a fixation in vertically moving mobile feeds.

One widely used application for eye-tracking research is understanding how consumers interact with information in digital platforms. Eye-tracking allows for the quantification of attention paid to crucial advertising elements, such as a brand logo. Brand attention is a critical part of advertising effectiveness. An ad that produces good attention and enjoyment of the creative, may still fail to generate brand attention and associate with the brand (Pieters et al., 2002). Therefore, the quantification of visual attention to branded elements is an important and sought-after measurement and is a suitable test case to study whether an eye-tracking solution has the necessary sensitivity and specificity to measure brand attention (Plassmann et al., 2012).

Mobile eye-tracking glasses allow the study of visual attention with minimum intrusiveness and are popularly used for consumer research. In the literature, this method has been used for studying the visual parameters during reading from different digital devices, including smartphones (Miranda et al., 2018). However, regardless of the manufacturer’s recommendations, to our knowledge, there are no examples of studies where the settings of fixation detection algorithms have been adjusted to the specific viewing conditions of stimuli presentation on a smartphone. We believe this leaves room for inaccurate research results for eye-tracking studies conducted on smartphone screens with vertically moving content. In this paper, we employ brand elements in advertising presented in mobile phone screens to investigate and suggest settings for smartphone-specific fixation detection algorithms and offer a framework for selecting the parameters for other use-cases.

This paper will first present the theoretical, practical, and empirical background for the selection of eye-tracking fixation detection algorithm settings, including presenting the need for mobile-specific recommendations for fixation filters and the parameters that are under consideration. As a case for this treatment, we will focus on the most abundantly used and dominant vendor, Tobii^1^, and their Pro Glasses 2 solution, alongside the challenges faced in measuring fixations on smartphones. After this, we will discuss a study designed to observe natural eye movements in mobile feed environments and compare performance of various filter options against human classification, as well as considering the validity and reliability of eye-tracking metrics. Here, we will use a model where we focus on advertising content presented on mobile phone screens. This is, to the best of our knowledge, the first study to report the results of a comprehensive and dedicated study on eye-tracking fixation detection algorithms for consumer behavior relevant behaviors on mobile phones. This work was supported by Facebook Inc and by the European Commission project RHUMBO – H2020-MSCAITN-2018-813234 TR.

## Background

### Operational Definition of a Fixation

Visual attention is inferred from patterns of eye movements, where visual information is acquired during brief periods when the eye remains relatively stable, which are called fixations. Eye-tracking systems record raw eye movement samples with specific gaze coordinates and timestamps at a specific frequency, and these raw gaze samples are usually filtered according to spatial and temporal parameters in order to calculate fixations. Filtering involves applying an event detection algorithm that eliminates the oculomotor events that do not meet the preset criteria from the fixation dataset (Holmqvist et al., 2011), but what constitutes a fixation can vary significantly depending on the characteristics of eye-tracking hardware and software parameters. The lack of consensus related to defining a fixation is further emphasized in a study by Hooge et al. (2017), which demonstrated that even though human classification of fixations is a common method for validating event detection algorithms, human coders apply different thresholds and selection rules, thereby arriving at different results. In the context of this paper, we use the term ‘fixation’ to signify a cluster of gaze samples during which the acquisition of visual information is likely to take place, and we suggest an improved approach for quantifying eye movement data for visual attention research on smartphones.

### Strengths of Measuring Fixations Instead of Relying on Raw Gaze Samples and Dwells

Mobile eye-tracking has been employed in various studies in dynamic real-life environments. In most of the cases, researchers analyze the recordings frame by frame and code the gaze behavior based on raw data samples. This means that instead of detecting and extracting fixations, the analysis of visual attention is based on dwells, i.e., gaze samples residing in the same area of interest (AOI). The problem with using raw gaze samples is that, in addition to eye movements that reflect the intake and processing of visual information, raw samples also include noise, such as saccades, drifts, tremors, and flicks. This implies that using raw gaze samples is likely to lead to an overestimation of visual attention allocated to the AOIs. On the one hand, when the same data is analyzed based on dwell durations rather than the sum of fixation durations, the values are approximately 20% higher (Holmqvist et al., 2011). As this increase in total viewing time applies to all AOIs, the conclusions based on the two different approaches may be fairly similar. However, in studies with high visual clutter (as is the case for digital media related stimuli), one of the metrics commonly used is the ratio or proportion of participants who gazed or fixated on an AOI (Percent Seen). When interpreting all raw gaze samples as indicators of an AOI having been seen, the inclusion of noise will distort the data and will likely lead to erroneous results. Because of this, it is most appropriate to apply a minimum threshold when calculating total viewing time in order to remove noise from the data.

Datasets of raw samples usually contain a substantial amount of ‘stand-alone’ gaze points that are located in the proximity of or between two target locations. As these gaze points have the duration too short to be reflective of information processing, they are usually considered noise and removed from the dataset. Studies employing mobile eye-tracking methodology in dynamic environments have used the cutoff point for dwells ranging from 100 ms (Gidlöf et al., 2017) to 120 ms (Visschers et al., 2010; Clement et al., 2013; Gidlöf et al., 2013), which aligns with the observation that human fixations generally last between 150 and 600 ms (Duchowski, 2007). However, it has also been shown that during reading tasks the fixation durations can be as short as 50–75 ms, and in visual search tasks, the range of fixation duration is greater, with the average fixation duration similar to reading tasks (Rayner, 2009). Since natural interactions with a digital media platform can be thought of as a free-viewing task that includes a combination of both reading and visual search (that is, of engaging information), the cutoff point for dwells should be set accordingly.

### The Need for Mobile-Specific Eye-Tracking Fixation Filters

One substantial challenge in applying the existing fixation detection algorithms to smartphone eye-tracking studies is that the conditions in which the default parameters for the fixation detection algorithm are developed can be markedly different from the study setup where the stimuli appear on a smartphone screen.

What is commonly overlooked about the Tobii I-VT Fixation Filter is that its default parameters were developed based on a stationary setup where participants were seated at a 65 cm distance from the screen that ranged from 43 to 61 cm in size (i.e., the resolution of 1280 × 1024 to 1920 × 1200 pixels) (Olsen, 2012). In contrast, the distance for viewing a smartphone is generally half of the viewing distance to a monitor. As an example of its application, in a study by Öquist and Lundin (2007), the distance from the smartphone was kept constant at 33 cm and smartphone screens are in the proximity of 13 cm, in contrast to the 65 cm distance and 43–61 cm screen size that the Tobii parameters were developed under. Furthermore, it is explicitly stated in Tobii’s white paper that the aim of their Fixation Filter development was to identify a set of default values that are suitable for the stationary eye trackers, which excludes head-mounted eye-tracking glasses, yet the fixation detection algorithm is widely used for studies utilizing Tobii Pro Glasses 2, nonetheless.

As an example of the challenges of using the Tobii I-VT Fixation Filter, the Tobii Studio User’s Manual v3.4.5 states:

“The I-VT fixation filter will fail to correctly classify eye tracking data recorded with Tobii Glasses even though the I-VT fixation filter may be enabled by default during analysis of snapshots. Tobii Glasses users should manually switch from I-VT fixation filter to Raw data filter. For information concerning limitations of the Raw data filter applied on Tobii Glasses recordings, please refer to the Tobii Glasses User manual.”

Despite this, many researchers continue to use Tobii Pro Glasses 2 hardware with the Tobii I-VT Fixation Filter, such as a study investigating visual parameters during reading from paper and various digital devices – smartphone, tablet, and computer screen (Miranda et al., 2018).

### Lack of Evidence for Mobile Filter Development and Usage

As previously mentioned, in a study by Miranda et al. (2018), mobile eye-tracking glasses were used to investigate visual parameters during reading from different modalities. Applying the default, I-VT Fixation Filter with the velocity threshold parameter of 30°/s, the authors suggested that similar criteria have been used also with other systems (e.g., Macedo et al., 2011). Mobile eye-tracking methodology has also been employed in various studies in dynamic real-life environments that rely on fixation data and only mention the software used (e.g., Tobii Studio), but do not discuss the parameters based on which the fixations were detected (Tonkin et al., 2011; Snyder et al., 2015). Also, the manual work of “experienced human coders” has been used for generating fixation data (Otterbring et al., 2016). There are also studies where no fixation detection algorithm is applied and the analysis of visual attention is based on dwells, i.e., gaze samples residing in the same AOI (Visschers et al., 2010; Clement et al., 2013; Gidlöf et al., 2017, 2013). A more ambiguous approach in the analysis of visual attention can be considered the “number of observations,” defined as “viewing an AOI until switching away” (Wästlund et al., 2015). Thus, it can be stated that there are no standardized or preferred approaches when it comes to the analysis of mobile eye-tracking data, and to the authors’ knowledge, there have not been any attempts of optimizing the parameters of the fixation detection algorithms to the viewing conditions or stimuli.

### Challenges Posed by Eye-Tracking on Mobile Phone Screens

#### Size and Distance of Display Impacts Saccade Length and Velocity

**FIGURE 1:**
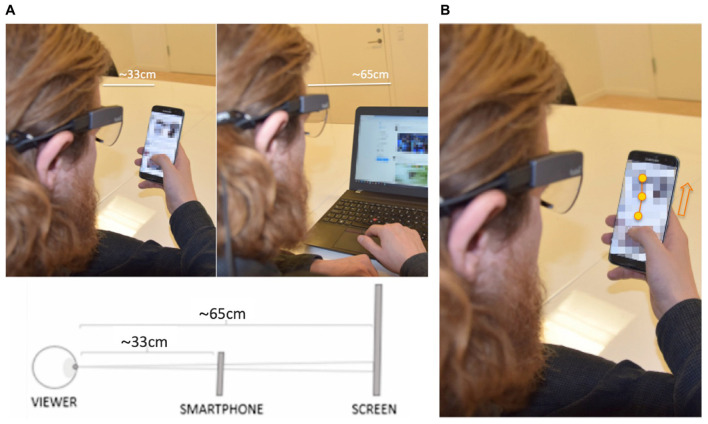
**(A)** Mock image of stimulus presentation on a smartphone screen versus a computer screen (obtained and published with consent). The default settings for the Tobii I-VT algorithm are based on a stationary setup with the viewing distance of approximately 65 cm. When looking at a smartphone screen the stimulus area is smaller and located at a closer distance, resulting in reduced saccade lengths and velocity of the eye movements. **(B)** Mock image of smooth pursuit while gazing at a dynamic stimulus (obtained and published with consent). The respondent is scrolling the screen while the gaze is fixated on a moving stimulus object, resulting in smooth pursuit. The eye-tracking system recognizes the movement of the eye and depending on the parameters of the fixation detection algorithm, may classify the gaze samples as saccades, excluding them from the fixation dataset.

The stimulus presentation method on a smartphone and, more specifically, the size of the display is likely to have an impact on saccade length and velocities of the eye movements. Namely, larger stimulus areas induce longer-distance saccades, which have been shown to generate higher velocities than shorter-distance saccades (Collewijn et al., 1988). Figure 1A visualizes the two viewing conditions with the stimuli presented on a smartphone screen versus a computer screen. A small stimulus display located at a close distance to the viewer induces shorter saccades with lower velocities, as well as more eye convergence, indicating that the Velocity-Threshold Identification (I-VT) fixation classification parameters need to be adjusted to the study setup.

If the velocity parameter value of the I-VT fixation detection algorithm is set too high, short saccades will be missed and fixations that should be separated are merged together. However, a velocity threshold set too low can lead to the fixations being split into a number of short fixations due to noise or short data losses (Olsen, 2012). This becomes especially important within a smartphone experience, where the smooth pursuit of vertically scrolling content could be mistaken for saccades and discarded if suboptimal settings are used.

#### Smooth Pursuit of Small, Moving Areas of Interest

One of the major problems associated with detecting fixations in mobile environments is that gaze data is recorded relative to the eye tracker coordinate system (i.e., head-mounted eye-tracking glasses), not that of the stimulus. This means that when the respondent’s eyes remain fixated on the stimulus while the respondent’s head or the stimulus is moving, the eyes are moving relative to the eye-tracker, resulting in vestibulo-ocular reflex or smooth pursuit. If the movement of the eyes exceeds the velocity threshold of Tobii’s I-VT Fixation Filter, these gaze samples are typically classified as saccades or unknown eye movements and excluded from the fixation data (Tobii Technology, 2019). Figure 1B explains the occurrence of a smooth pursuit while the respondent is scrolling the screen of a smartphone.

#### Incorrect Fixation Detection Can Create Gaze Mapping Issues

In relation to eye-tracking research that involves viewing mobile devices, there are stationary solutions where the restrictive setup allows for automated mapping of gaze locations. As an example, Öquist and Lundin (2007) used a setup with chinrest and goggles, and a smartphone that was kept at a fixed position. A downside for the setup where the participants’ head is held in a fixed position is that it substantially reduces ecological validity. Researchers who prioritize investigating natural viewing behavior with minimum intrusiveness are likely to prefer head-mounted eye-tracking sensors, which inevitably require an additional phase of data pre-processing, namely, mapping the gaze positions from a video recording to reference images. This work is typically done by human coders, and considering that a device with the sampling frequency of 50 Hz records a new gaze sample at every 20 ms, the number of man-hours required to pre-process the data for even a small study, quickly becomes overwhelming and expensive. This issue has also been reported in the literature – Pfeiffer et al. (2014) utilized 30 Hz eye-tracking glasses and reported that the manual coding of the eye movements of 20 respondents in four decision situations required about 80 h. The manual coding for the dataset presented in this paper alone required 321 labor hours, or roughly 2 h per respondent to code all raw data samples from 152 individuals. This illustrates an obstacle for running studies with larger samples, which, in turn, can have a negative impact on the reproducibility of the findings. Until there are solutions for automated annotations, one way to reduce that manual workload is by using a fixation detection algorithm, which significantly reduces the amount of data prior to remapping the eye movements and is one of the motivations for this study.

### Existing, Widely Adopted Mobile Eye-Tracking Fixation Detection Algorithms and Approaches

Because Tobii Pro Lab Filters are the most widely used fixation detection algorithms in the body of research we examined and discussed above, this article will begin by focusing on the settings that can be manipulated in those filters and the function of each, in order for the findings presented in this paper to have the most relevance for industry applications. Depending on the sampling rate of the eye tracker, raw eye movement samples with specific gaze coordinates and timestamps are recorded at a specific frequency (for Tobii Pro Glasses 2, the sampling rate is 50 Hz). Tobii Pro Lab uses a fixation detection algorithm called the Velocity-Threshold Identification (I-VT) Fixation Filter (e.g., Salvucci and Goldberg, 2000; Komogortsev et al., 2010). The function calculates the angular velocity for each data point and depending on the threshold value classifies data points as being part of a fixation or a saccade (Olsen, 2012).

Tobii Pro Lab has three preset data processing functions that can be applied to the eye movement data, as presented in Table 1.

**TABLE 1:** The three preset data processing functions of Tobii Pro Lab. Source: adapted from Tobii Technology (2019).

Preset function	Raw Gaze Filter	Fixation Filter	Attention Filter
Description:	Extracts raw gaze samples with three pre-processing functions	Applies I-VT fixation classifier algorithm with the velocity threshold of 30°/s	Applies I-VT fixation classifier algorithm with the velocity threshold of 100°/s
Data composition:	Data includes all gaze samples (including all noise)	Excludes smooth pursuit and vestibulo-ocular reflex (VOR)	Includes smooth pursuit, VOR and 10–15% of saccades
Attention allocated to the stimulus:	Overestimated	Slightly underestimated	Slightly overestimated
Best suited for:	Studies focusing on mechanisms of vision and dwells	Controlled studies with only fixations and saccades	Mobile environments with movement

After having applied the data processing functions to the eye movement data, gaze samples or fixations are overlaid on a video recording. In order to aggregate gaze data and calculate eye-tracking metrics, the gaze samples or fixations need to be remapped from scene recordings to a reference image (Tobii Technology, 2019), which, as mentioned earlier, can take a substantial amount of time to map.

### Tobii Pro Glasses 2 and the Fixation Detection Algorithm

The Tobii Pro Glasses 2 rely on an absolute measurement of pupils and use corneal reflection and the dark area of the pupil to detect the position of the eyes. The compensation for parallax is automatic and the method is based on binocular eye-tracking technique with 50 or 100 Hz frequency (Tobii, 2015).

Tobii Pro Lab software allows the use of preset settings for fixation detection algorithms, but also to create new fixation filters (Tobii Technology, 2019). In total there are seven different parameters that can be adjusted, including Gap fill in, Eye-selection, Noise reduction, Window length, Velocity threshold, Merging adjacent fixations, and Discarding short fixations.

### Study Overview

In this study we compared the output gaze metrics produced with a commonly used fixation detection algorithm against the output of a manual mapping of raw gaze on AOIs. A difference between these two techniques is the order of mapping raw gaze data on to AOIs. The Tobii I-VT Fixation Filter calculates fixations prior to mapping gaze to AOIs, meaning any gaze data that is not counted as a fixation does not get mapped on to an AOI. Depending on the settings, this can lead to the possibility of miscounted fixations when the eye is following a moving target. Additionally, fixations on two nearby AOIs may be averaged together, making it possible for the coordinates of a fixation to be uncounted in gaze metrics if the fixation coordinates fall outside of the AOI boundaries. In contrast, the ground truth in this study relies on manual gaze mapping from raw eye gaze data, which maps all raw gaze data coordinates to AOIs prior to filtering for fixations or calculating gaze metrics at the AOI-level for moving targets. This enables researchers to capture more accurate metrics at the AOI-level — such as the percentage of people who saw the AOI (Percent Seen) or how long each person spent looking at the AOI (Total Fixation Duration)— when the eye is following a moving target. As noted in section “Operational definition of a fixation,” human classification of fixations is a common method for validating event detection algorithms (Hooge et al., 2017), therefore, human classification is what we have employed for validation in this study.

In this paper, we utilized a common scenario where eyes follow moving targets, a vertically scrolling mobile phone feed, and compare outputs of the Tobii I-VT Fixation Filter to the outputs of manual mapping of raw gaze data on AOIs with a fixation defined as >60 ms consecutive gaze points within an AOI, based on the appropriate fixation length of reading and visual search described above in section “Strengths of measuring fixations instead of relying on raw gaze samples and dwells.”

The purpose of utilizing existing widely available hardware and software, as well as the application to mobile feed environments, was to ensure that the results were readily applicable to industry use-cases. In order to observe velocities and patterns of natural eye movements that occur when people view a vertically scrolling mobile feed, and for the results of the study to be applicable to future research on advertisements in a mobile social media feed, we conducted this study in a self-paced, natural environment with insertion of 10 controlled test stimuli into the feed. While this paper focuses on capturing natural behaviors in feed environments, future research is recommended to explore the spatial accuracy of eye-tracking measurement on highly controlled target stimuli on mobile phones.

Prior to the present study, we conducted studies to explore a broader space of filter settings options, and thereby to narrow down the filter settings for the present study (see Supplementary Materials). The goal of this study was to compare 56 possible fixation filter settings to determine which settings most closely align with human classification of fixations and gaze metrics in a social feed with vertical motion. The output is intended to inform settings for future research that examines eye-tracking on advertisements in a mobile social feed environment.

## Materials and Methods

This study was performed in accordance with the Declaration of Helsinki. Neurons Inc. follows the rules and laws of the Danish Data Protection Agency^2^. Neurons’ data protection policy also follows the European Union law of the General Data Protection Regulation^3^, as well as the ethical regulations imposed by the Neuromarketing Science and Business Association, Article 6^4^. Each person’s biometric data, survey responses, and other types of data were anonymized and only contained the log number as the unique identifier. No personally relevant data can be extracted from the log number.

Tobii Pro Glasses 2 50 Hz were used for binocular data collection and Tobii Pro Controller (v.1.7.6) software was used for recording the data. Participants were exposed to 10 controlled static ads in their own Facebook feed environment. The ads were inserted using Neurons Inc’s ad-insertion solution to provide a true to nature experience for the participants. All participants were exposed to all ads in a fully randomized order, with four organic posts in-between each ad. Stimuli were presented on Samsung Galaxy S7 Edge smartphones with 13 cm screens. The study took place in New York City in a quiet room with consistent overhead LED and incandescent lighting conditions of approximately 300 lux, with each participant monitored 1:1 in an individual room.

The study was conducted with 172 respondents with normal or corrected-to-normal vision and no history of neurological or psychiatric disease. Participants were permitted to wear contact lenses during the session, but glasses-wearers were excluded from the recruitment sample. Participants recruitment criteria included an even split of males and females, a mix of ethnicities, above the age of 18. The participants were individually seated at a table and held the smartphone in their hands throughout the session. Only one participant was in the room during a session. The eye-tracker was individually calibrated by participants focusing their gaze on the center of a target calibration card. The accuracy of the recording was then tested via live view function, where participants were instructed to look at static target objects displayed on the smartphone screen, which was visually validated by moderator.

After ensuring accurate calibration, the study session was started. The participants were instructed to maintain an optimal viewing posture and refrain from moving the phone during the viewing tasks. The optimal viewing posture was measured to 30–35 cm from the glasses to the phone. People held the phones themselves to provide a normal reading/browsing experience, while going through the social feed. The experimenter monitored the participant’s viewing posture for the correct distance from glasses to phone and would instruct the participant to return to the desired distance as needed. All instructions related to the viewing tasks were presented in text form on a laptop screen. After the viewing tasks were completed, the accuracy of the gaze recording was again tested based on static target objects. The participants were then thanked for their time and could leave the testing area.

**FIGURE 2:**
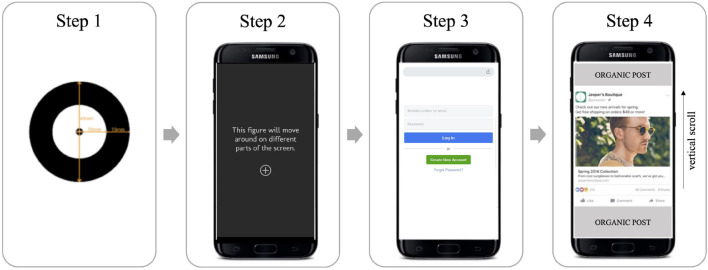
An illustration of the participant study flow. **Step 1**: The participant goes through an eye-tracking calibration scenario. **Step 2**: The eye-tracking calibration is validated with a 5-point validation scenario presented on a smartphone screen. **Step 3**: The participant logs in to their social media account and opens the main feed. **Step 4**: The participant browses their feed in a self-paced session, where the ads have been inserted every 5th post.

The study consisted of a natural browsing session in each participant’s own social media feed. Participants were instructed to log in to their social media account and browse through their feed as they normally do. Ten individual static ads were inserted into each person’s feed using an in-house ad insertion software running on Android 8.0.0 version. Prior internal pilot research showed that ten advertisements was the maximum number of ads that could be presented within the study time window while maintaining attention from participants. Ten ads were chosen to provide a larger sample and variance in the type of ads ensuring ecological validity for future application of the filters. A visual representation of this task can be seen in Figure 2. The duration of the sessions varied due to the natural exposure setting. A total of 10 advertising stimuli were presented in the feed with four organic posts in between each ad, with the order of ad presentation balanced across groups. Duration of ad exposures varied due to the natural exposure setting.

### Mobile Fixation Filter Development

#### Defining a Fixation

The purpose of this study is to investigate the impact of different parameters of the above fixation detection algorithms on eye-tracking metrics of natural viewing behavior. Establishing an optimal fixation definition for a research study requires several considerations, such as an investigation into various aspects of natural viewing behaviors in a specific setting. It was previously established that for a study that involves interacting with digital media, the cutoff point for minimum dwell length should be set in accordance with the characteristics of the viewing behavior. Based on the argumentation that during reading and visual search tasks the fixations can be markedly shorter than fixations during scene perception, that is, as short as 50–75 ms (Rayner, 2009), we consider the cutoff point 60 ms as the optimal setting for minimum dwell length. As we are focusing on a device that has a sampling frequency of 50 Hz, this corresponds to three consecutive gaze samples in an AOI.

Considering the problems with the standard preset filters, there are strong arguments in favor of using dwells with the cutoff point of 60 ms as a more objective and accurate approach for measuring eye movements. This, however, preserves the problem of extensive resources that are needed for remapping raw gaze samples. As previously mentioned, the number of man-hours can be reduced significantly if the raw gaze samples are filtered with a fixation detection algorithm prior to remapping the eye movement data from video streams to reference images.

#### Selecting the Fixation Filter Parameters to Be Modified

Based on an investigation of relevant literature, together with qualitative observations of how the changes in different parameters affect the data output, it was decided to focus on and modify three parameters: Velocity threshold, Merging adjacent fixations, and Discarding short fixations. The following paragraphs explain the argumentation behind these choices.

#### Velocity Threshold

Velocity threshold is the parameter value based on which each data point is classified as being a part of a fixation or a saccade, whereas a fixation comprises an unbroken chain of raw data samples that have the angular velocity below the velocity threshold (Tobii Technology, 2019). It is known that larger stimulus areas induce longer saccades, which have shown to generate higher velocities than short saccades (Collewijn et al., 1988). This implies that when viewing a smartphone screen, the saccades have lower angular velocity, and the threshold for velocity needs to be set lower, to achieve more sensitive measures of eye movements and more granularity in the data. A velocity threshold set too high is likely to miss short saccades, and shorter fixations that should be considered as separate are merged into long inaccurate fixations. Combined with the qualitative insights of how changing the velocity threshold affects data output, we decided to test all thresholds between 5 and 15°/sec, and also include the thresholds 20, 25, and 30°/s for estimating the effect of increasing the velocity threshold on the validity of the data. Angular velocity values above 30°/s were not chosen as the original Tobii Fixation Filter is based on 30°/s and it has been shown in earlier research that this setting was too inclusive for larger AOIs and too exclusive for smaller AOIs.

#### Merging Adjacent Fixations

The function of merging adjacent fixations is aimed to correct for errors caused by noise and disturbances where a single fixation is split into multiple short fixations located close together (Tobii Technology, 2019). When enabled, this setting by default merges the fixations together if they are maximum 0.5 degrees apart and occur in a maximum 75 ms time frame. When stimuli are presented on a smartphone screen, small AOIs can be located in close proximity. In such cases the function of merging adjacent fixations may lead to reduced accuracy. Furthermore, not merging adjacent fixations will lead to a data output that is closer to raw gaze points, and thereby also closer to actual eye movements. For this reason, we decided to test the algorithms with the function of merging adjacent fixations both enabled and disabled.

#### Discarding Short Fixations

The function of disregarding short fixations is aimed to remove incorrectly classified fixations that are too short for actual information acquisition and processing (Tobii Technology, 2019). When enabled, this setting, by default, removes all fixations that have a duration below 60 ms. However, natural viewing of digital media stimuli on a smartphone screen often involves scrolling, which in turn leads to smooth pursuit. This means that the participant is keeping the eyes fixated on an AOI while the stimulus is moving, but because of the movement of the eyes, fixation detection algorithms may disregard a substantial proportion of these gaze points, as illustrated in Figure 1B. To avoid this loss of data, it may be useful to keep the short fixations in the dataset. Disabling this setting adds more granularity to the data and will make the data output better reflective of the actual eye movements. For this reason, we decided to test the algorithms with the function of discarding short fixations both enabled and disabled.

The decision to focus only on the three above-mentioned parameters is that we considered these functions to have the most significant impact on the validity of the fixation data output, as compared to our target measure of dwells of 60 ms – which we refer to as Raw60 in this paper. All other parameters were kept at their default value, and their potential impact is out of the scope of this study. We acknowledge that adjustments to other parameters are also likely to affect the data output, but as we discuss next, we did not see the utility in testing their impact.

#### Parameters That Were Kept Constant

Tobii has developed three different pre-processing approaches, where two of them apply the I-VT fixation classifier algorithm to detect fixations. (*Additional details about the Tobii preset data processing functions are included in Supplementary Material*). The first option, Raw Gaze Filter, allows extracting raw gaze samples rather than fixations, but nevertheless, it applies three pre-processing functions to the data: gap fill-in or interpolation, eye selection, and noise reduction based on moving median approach. The same three functions are also applied to the two preset data processing functions that apply the I-VT fixation classifier, Fixation Filter, and Attention Filter. Considering that these three pre-processing functions are by default applied even to raw gaze data, it was decided to keep these parameters unchanged.

Another parameter that was decided to keep unchanged is the window length of the I-VT fixation classifier. By default, it is set to 20 ms, which corresponds to the sampling frequency of the eye-tracker used. Thus, it is not possible to set this value any lower, and considering that, given the stimulus presentation method and characteristics of the stimuli, the objective is to determine the settings that capture the eye movements with more sensitivity and higher granularity, we do not see the value in increasing the window length and thereby make the measures more robust.

### Data Analysis

After collecting all the data, the marking process was executed by a team of human coders from Neurons Inc using the Tobii Pro Lab Software (V 1.79.10518), where each raw gaze point was mapped between the glasses coordinates and the reference frame image in the field of view, otherwise called the Scene Camera. We refer to this process as Gaze Mapping.

**FIGURE 3:**
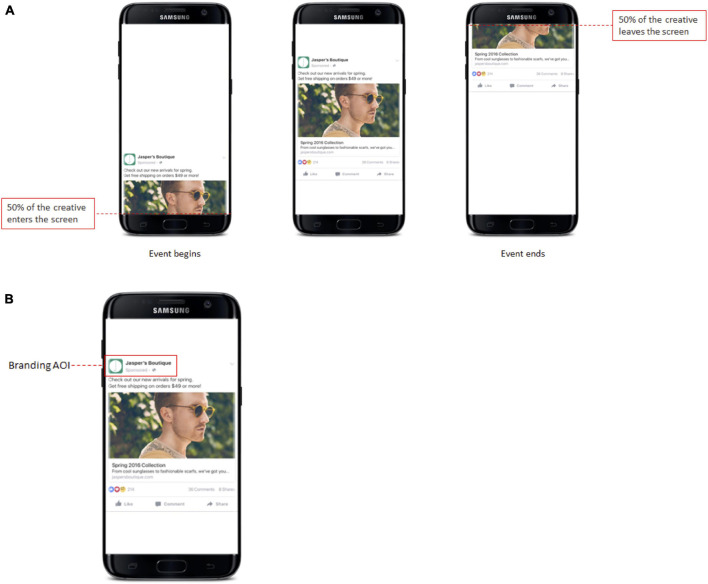
**(A)** A visualization of annotating the onset and endpoint of a stimulus as an example of placing the beginning and end of events. **(B)** An example of Branding AOI.

To specify the important parts of the recording we defined and logged stimuli events in each of the recordings. A stimulus event was logged as “on screen” from when 50% of the stimulus (Feed post) entered the screen until 50% of it left the screen (see Figure 3A).

Tobii Pro Glasses 2 produces eye gaze data mapped to a coordinate system relative to the wearable eye tracker and the recorded video, not to static objects of interest in the environment around the participant wearing the eye tracker. The collected eye-tracking data needs to be mapped on to objects of interest and into a new coordinate system with its origin fixed in the environment around the participant (Tobii Technology, 2019). Using the manual mapping function of the Tobii Pro Lab, our coders mapped the exact location of each gaze point on the static snapshot of the stimulus.

The *branding* AOI was drawn around the profile picture and profile name area on each of the 10 stimuli (Figure 3B). Gaze Mapping was done on raw gaze data and the fixation data output was exported based on 56 different combinations of algorithm settings.

Due to the importance of precision in the study, we excluded 16 participants having less than three (out of five) hits on the calibration verification task. The final sample thus included 156 participants (age range: 19–72, mean age: 36.9, Std Dev:12.54, 75 females).

#### Selecting Metrics of Interest

This analysis focuses on the three main eye-tracking metrics Total Fixation Duration (TFD) which is defined as the accumulated duration of fixations on an AOI, Time to First Fixation (TTFF) or the time it takes before an AOI is seen from the moment that 50% of the feed post enters the screen (Beginning of the event), and the percentage of participants that have at least 1 fixation (= “seen”) on a stimulus or an AOI (Percent Seen).

Tobii Pro Lab software allows users to use preset settings for fixation detection algorithms, but also to create new ones (Tobii Technology, 2019). In all cases, the data extracted based on different algorithm settings is compared to manually mapped gaze data output, which we refer to as Raw60. To make the Raw60 metrics, the raw data has been preprocessed so that the gaze has to remain on an area of interest (AOI) for a minimum of 60 ms (3 gaze points) in order for it to be classified as “fixation” on that AOI.

Based on these parameters, the total number of setting combinations was 14 × 2 × 2 = 56, based on the functions of 14 different velocity thresholds, merging/not merging adjacent fixations, discarding/not discarding short fixations.

#### Measuring the Error of Different Metrics Under 56 Proposed Test Filters

Because our goal was to select an improved fixation detection algorithm for vertically moving content presented on a smartphone screen, it was important to consider the impact our 56 proposed fixation detection algorithms (Test filters) had on the error of our metrics of interest. We first summarized the metrics of interest in relation to each of the stimuli and all AOI. The data covered all the three metrics of interest [Total Fixation Duration (TFD), Time to First Fixation (TTFF)] and viewer ratio (Percent Seen) and allowed to compute the differences between the outputs of each of the 56 Test filters and the benchmark of Raw60 (i.e., dwells with the cutoff point of 60 ms).

Using Raw60 as the benchmark, we distinguished between overestimation and underestimation of the TFD. Overestimation of TFD (false positives) occurs when the fixation detection algorithm merges together a number of gaze points that landed outside the area of interest with gaze points inside the area of interest, bringing the TFD higher than possible. This represents an indicator of insensitivity and inaccuracy of the algorithm and sought to be kept as low as possible. The underestimation (false negatives), in contrast, reflects a situation where the TFD based on Raw60 is lower for the Test filter, showing a “loss” in data. The underestimation is seen as less impactful, when considering that Raw60 is likely to include a small share of gaze points that represent saccades or other types of noise. The improved settings of the fixation detection algorithm are expected to result in overestimation close to zero, with underestimation as low as possible.

For the metric TTFF, our objective was to minimize the difference between the TTFF (Raw60 ms) and the TTFF(Test filter). The error magnitude was computed as the absolute difference between the two variables. For the ratio of viewers who have seen an AOI (Percent Seen), on the participant level the data obtains a binary value depending on whether there are eye movements recorded on the AOI (“1”) or not (“0”). It is desirable that the fixation filter with the optimized settings leads to the Percent Seen values that correspond as closely to the Percent Seen based on Raw60 as possible, and accordingly, the errors were defined as mismatches between the Seen(Raw60) and Seen(Test filter). The description of all error components is shown in Table 2.

**TABLE 2:** Parameters for assessing the differences in the outputs of the benchmark (Raw60) and the adjusted fixation detection algorithms (Test filters).

Error component	Description
Overestimation of Total Fixation Duration (TFD)	The value by which the TFD(Test filter) exceeds the TFD(Raw60)
Underestimation of Total Fixation Duration (TFD)	The value by which the TFD(Raw60) exceeds the TFD(Test filter)
Error in Time to First Fixation (TTFF Error)	The absolute value of the difference between the TTFF(Raw60) and TTFF(Test filter)
Error in the ratio of participants viewing the Area of Interest (Percent Seen Error)	Mismatch between the Seen(Raw60) and Seen(Test filter)

#### Understanding the Error on a Branding Area of Interest

**FIGURE 4:**
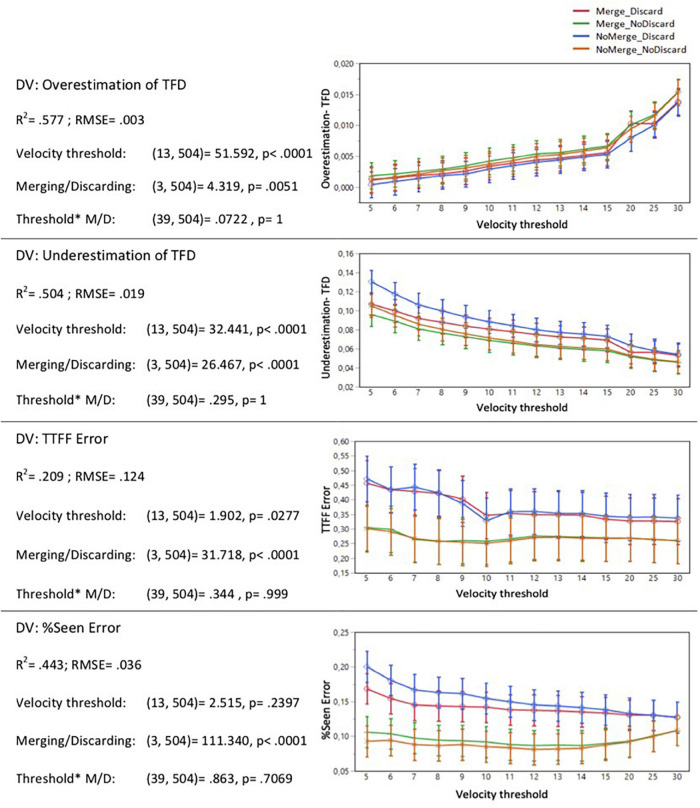
Error components of different eye-tracking metrics as a function of different fixation detection algorithm parameters for AOI “Branding”.

As the next step, we computed the mean values of the error components on the Stimulus and AOI level by averaging the data across all participants. To assess how the algorithm parameters influenced the error components for the AOI ‘Branding,’ we ran a linear least squares regression based on REML with the error component as the dependent variable and Discarding/Merging, Velocity threshold, the interaction between the two as model predictors. Figure 4 presents how the different parameters of the Test filters influenced each of the error components.

#### Determining the Precision of Each Test Filter

**FIGURE 5:**
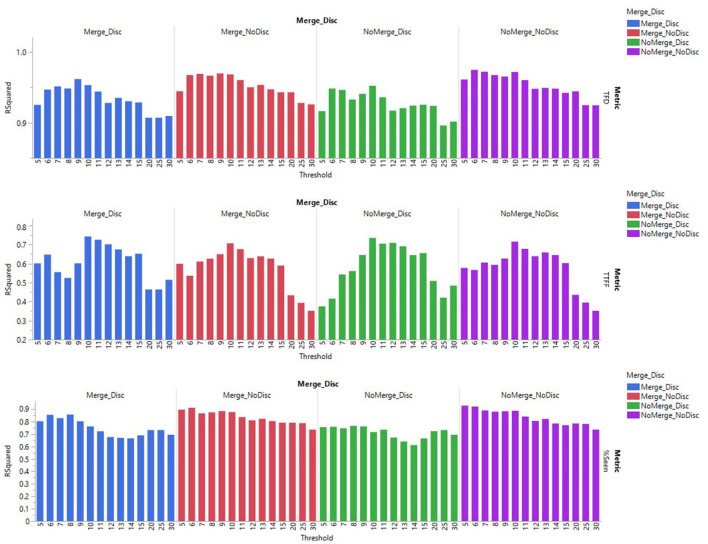
Coefficient of determination for each of the Test filters and the benchmark of Raw60.

To assess the degree to which the output of different Test filters correlated with the output of the benchmark Raw60 output, we ran a linear regression between the mean values of each of the metrics (TFD; TTFF and Percent Seen) on the Stimulus and AOI level (i.e., averaged across all participants). Focusing on the AOI ‘Branding’, we used the coefficient of determination (*R*^2^) as the indicator of how well each of the Test filters performs, as compared to the benchmark output. The results are presented in Figure 5.

#### Reliability of the Metrics

**FIGURE 6:**
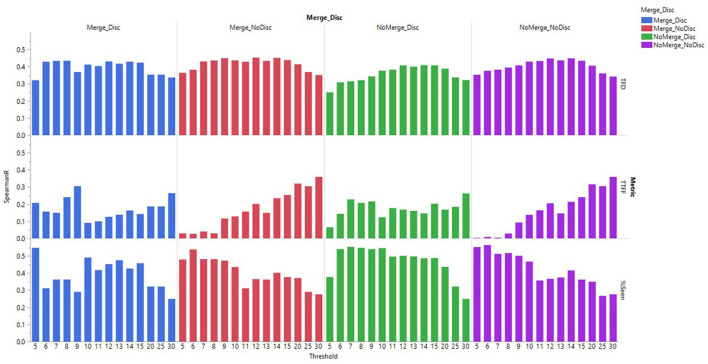
The mean Spearman R scores across 1000 iterations of randomly split-half samples for different Test filters.

In addition to validity, we assessed the effect of the fixation detection algorithm settings on the reliability of the data (i.e., the consistency of the fixations across participants exposed to the same stimuli). Inter-subject reliability is relevant, as we know that stimuli should drive eye-tracking response, and inconsistency between participants can thus be a sign of measurement error or noise. To measure the inter-subject reliability of each stimulus, participant data was randomly split into two halves (78 participants in each half). For each half, we calculated the eye-tracking metric, and subsequently the Spearman correlation across ads for Branding AOI, using Spearman’s Rank Correlation (Spearman *R*). This was run as a bootstrapping procedure with 1000 repetitions, each time taking a new random split from the total pool of participant data. Across all iterations and for each of the metrics, we calculated the mean Spearman R score for each of the Test filters (Figure 6).

## Results

### Descriptive Statistics

Ten advertising stimuli were inserted in participants’ own social media feeds. Participants were exposed to 8 of the 10 ads on average (mean: 8.25, Std Dev: 2.5, min:2, median: 10, max: 10). Duration of ad exposures varied due to the natural exposure setting, with an average of 2–3 s of view time per ad (mean: 2.67 s, Std Dev: 2.52 s). The total browsing time for the session varied due to the natural exposure, with an average session time of approximately 5 min (mean: 327.10 s, Std Dev: 115.86 s).

### The Impact of the Fixation Detection Algorithm Parameters on the Error Components of Different Metrics

As visible in Figure 4, there was a significant positive relationship between the velocity threshold and the overestimation of TFD, and a significant negative relationship between the velocity threshold and the underestimation of TFD. This means that as the velocity threshold is increased, the test filter picks up more gaze points, but there is also a higher risk of obtaining inflated values of TFD. Thus, when optimizing the parameters for small AOIs such as the branding element of a sponsored content, there appears to be a tradeoff between the sensitivity of the algorithm (as indicated by the overestimation of TFD) and the underestimation of the total viewing time.

The Test filters with the function of ‘discarding short fixations’ disabled (marked with green and yellow lines in Figure 4) lead to decreased underestimation of TFD and lower error values for TTFF and Percent Seen. The differences are most drastic for the error in TTFF and Percent Seen.

While the impact of the algorithm parameters on error components allows assessing the directionality and magnitude of the effects, the choice of the optimized parameters often involves a tradeoff, and should, therefore, reflect the objective of the study and the characteristics of the stimuli. To directly assess how well each of the Test filters performed relative to the benchmark Raw60, we ran correlations on the outputs of different metrics, as described in the following section.

### Correlations Between the Output of the Test Filters and the Benchmark to Determine Filter Accuracy

The top graph in Figure 5 presents the correlation of determination or R^2^ values for different Test filters and the benchmark Raw60 for the metric TFD. Overall, the coefficient of determination values is the highest for the setting of NoMerge_NoDiscard with velocity threshold 6°/s (*R*^2^ = 0.975) and thresholds 7 and 10°/s (*R*^2^ = 0.972). Across all combinations of settings of merging adjacent fixations and discarding short fixations, it is evident that there was a decline in *R*^2^ values as the threshold was increased above 10°/s.

A one-way ANOVA comparing the *R*^2^ values across all thresholds revealed a significant difference between the four different types of settings of merging adjacent fixations and discarding short fixations, *F*(3,164) = 11.682, *p* < 0.0001. Table 3 presents the mean TFD values and confidence intervals for different settings, as well as pairwise comparisons with significant differences.

**TABLE 3:** *R*^2^ of TFD, TTFF, and Percent Seen – Mean values and standard errors of settings of merging adjacent fixations and discarding short fixations, and significantly different pairwise comparisons based on Tukey–Kramer HSD test.

Metric	Setting	Mean	Standard error mean		Mean	Standard error mean	HSD threshold	*p*-value
TFD	Merge_NoDisc	0.973	0.017	NoMerge_Disc	0.951	0.023	0.0103	**<0.0001**
	NoMerge_NoDisc	0.973	0.017	NoMerge_Disc	0.951	0.023	0.0103	**<0.0001**
	Merge_NoDisc	0.973	0.017	Merge_Disc	0.960	0.003	0.0017	**0.0174**
	NoMerge_NoDisc	0.973	0.017	Merge_Disc	0.960	0.003	0.0017	**0.0174**
TTFF	NoMerge_NoDisc	0.771	0.024	NoMerge_Disc	0.596	0.030	0.0852	**<0.0001**
	Merge_NoDisc	0.770	0.024	NoMerge_Disc	0.596	0.030	0.0844	**<0.0001**
	NoMerge_NoDisc	0.771	0.024	Merge_Disc	0.681	0.019	0.0003	**0.0490**
	Merge_NoDisc	0.770	0.024	Merge_Disc	0.681	0.019	–0.0005	0.0519
Percent seen	Merge_NoDisc	0.797	0.018	NoMerge_Disc	0.657	0.028	0.0103	**0.0003**
	NoMerge_NoDisc	0.795	0.020	NoMerge_Disc	0.657	0.028	0.0103	**0.0004**
	Merge_NoDisc	0.797	0.018	Merge_Disc	0.696	0.028	0.0017	**0.0162**
	NoMerge_NoDisc	0.795	0.020	Merge_Disc	0.695	0.028	0.0017	**0.0184**

A one-way ANOVA comparing the *R*^2^ values across all thresholds revealed a significant difference between the four different types of settings of merging adjacent fixations and discarding short fixations, *F*(3,164) = 11.715, *p* < 0.0001. Table 3 presents the mean TTFF values and confidence intervals for different settings, as well as pairwise comparisons with significant differences.

Finally, the bottom graph in Figure 5 visualizes the correlation of determination values for the ratio of participants who viewed the AOI – Percent Seen. Overall, *R*^2^ values were the highest for the setting of NoMerge_NoDiscard with velocity threshold 5°/s (*R*^2^ = 0.929), threshold 6°/s (*R*^2^ = 0.921), and the setting Merge_NoDiscard threshold 6°/s (*R*^2^ = 0.911). Across all combinations of settings of merging adjacent fixations and discarding short fixations, *R*^2^ values tended to be higher for velocity thresholds up to 10°/s, as compared to thresholds 11°/s and above.

A one-way ANOVA comparing the *R*^2^ values across all thresholds revealed a significant difference between the four different types of settings of merging adjacent fixations and discarding short fixations, *F*(3,164) = 8.833, *P* < 0.0001. Table 3 presents the mean Percent Seen values and confidence intervals for different settings, as well as pairwise comparisons with significant differences.

Comparing the data output to the scene recordings, it was found that velocity thresholds in the proximity of 10°/s provided the closest reflection of the actual eye movements. It was found that while 5°/s threshold sliced fixation data to overly small clusters of gaze points, the threshold of 15°/s merged gaze movements over several AOIs into long ambiguous fixations. Accordingly, these two values were decided to be taken as the two border conditions, implying that all discrete velocity values ranging from 5 to 15°/s would be tested as algorithm parameters.

**FIGURE 7:**
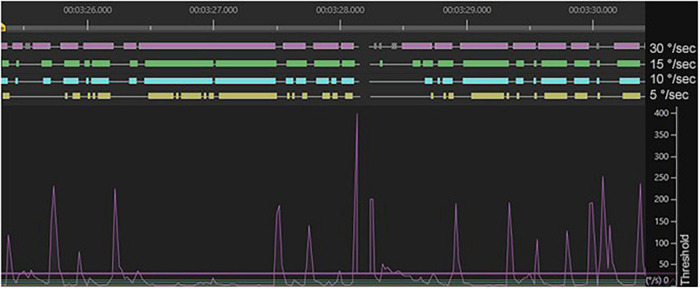
Fixation data output as the velocity threshold is modified. The angular velocities of the eye movements (vertical axis) are captured over several seconds of natural viewing behavior (horizontal axis) and the horizontal bars above the graphs signify the chains of gaze points that are classified as fixations.

Figure 7 visualizes the fixation data output when the velocity threshold was set to 30°/s (purple), 15°/s (green), 10°/s (blue), and 5°/s (yellow). Each horizontal bar signifies a chain of gaze points that are classified as a fixation.

### Reliability of the Metrics

Figure 6 presents the correlation of determination or *R*^2^ values for different Test filters and the benchmark Raw60 for the metric TFD. Overall, the coefficient of determination values was the highest for the setting of NoMerge_NoDiscard with velocity threshold 6°/s (*R*^2^ = 0.975) and thresholds 7 and 10°/s (*R*^2^ = 0.972). Across all combinations of settings of merging adjacent fixations and discarding short fixations, it is evident that there was a decline in *R*^2^ values as the threshold was increased above 10°/s.

Figure 6 presents the mean scores of Spearman R across 1000 iterations of randomly split-half samples for different Test filters. For TFD metric, the Spearman R score was highest for the setting NoMerge_NoDiscard and thresholds 12 and 14°/s and also Merge_NoDiscard with thresholds 9, 12 and 14°/s (Spearman *R* = 0.45).

A one-way ANOVA comparing the Spearman *R* values across all thresholds, revealed a significant difference between the four different types of settings of merging adjacent fixations and discarding short fixations, *F*(3,56) = 6.184, *p* = 0.0011. Table 4 presents the mean values and confidence intervals for different settings, as well as pairwise comparisons with significant differences.

**TABLE 4:** Spearman R of TFD – mean values and confidence intervals of settings of merging adjacent fixations and discarding short fixations, and significantly different pairwise comparisons based on Tukey HSD test.

Setting	Mean	Lower 95% CI	Upper 95% CI	Setting	Mean	Lower 95% CI	Upper 95% CI	p-value
*Merge_NoDisc*	*0.418*	*0.396*	*0.439*	*NoMerge_Disc*	*0.356*	*0.334*	*0.377*	*0.0009*
*NoMerge_NoDisc*	*0.404*	*0.383*	*0.426*	*NoMerge_Disc*	*0.356*	*0.334*	*0.377*	*0.0119*
*Merge_Disc*	*0.397*	*0.375*	*0.418*	*NoMerge_Disc*	*0.356*	*0.334*	*0.377*	*0.0446*

## Discussion

### Study Aim

The goal of this study was to understand the performance of existing, commonly used fixation filters for applications of measuring eye gaze on vertically scrolling smartphone screens, and to identify combinations of parameters that most closely align with human classified fixations, called Raw60 above. In addition to developing filters that are valid and reliable for smartphone applications, this study offers a framework that can be leveraged for future filter development as can be applied to many use-cases.

The focus of this study has been on measuring branding elements of advertising presented in vertically moving feed environments. These elements are not only necessary for establishing awareness of a brand, but also increasing brand salience, and are frequently desired to be understood by advertisers who undertake eye-tracking research for quantifying brand attention.

### Main Findings

Since there is no standard approach for detecting meaningful eye movements, and as it has been shown that event-detection algorithms perform poor for dynamic stimuli (Andersson et al., 2017), a number of studies using mobile eye-tracking glasses have used dwells with a specific cutoff point to analyze eye movements. Using dwells with the cutoff point of 60 ms as the benchmark, our objective was to determine the fixation detection algorithm parameters that would lead to the data output equivalent or as similar as possible to the benchmark in a setting where small areas of interest are presented on the screen of a smartphone. We modified three parameters of the Tobii I-VT fixation filter (velocity threshold, merging adjacent fixations, discarding short fixations) and investigated how different combinations of parameter settings measure against the data output based on Raw60.

To understand how different fixation detection algorithm parameters affected the data output, and more specifically, the differences between the data output based on the benchmark of Raw60 and different Test filters, we first looked at the error components of different eye-tracking metrics by running several linear regressions with algorithm parameters as model predictors and the error component as the response variable. The data revealed that for the TFD metric, increases in velocity threshold led to an increase in overestimation of viewing time. For the error component of underestimation of the viewing time, the relationship between the two variables was the opposite. This implies that there is a tradeoff – if a lower velocity threshold is chosen, the algorithm is more sensitive and thereby less likely to overestimate the viewing time, but, concurrently, picks up fewer gaze points and is more likely to underestimate the viewing time. The data also revealed that test filters with the function of discarding short fixations disabled lead to decreased underestimation of the viewing time but performed worse for the overestimation error component. The choice of the optimized settings, therefore, depends on whether it is more important to prioritize minimizing the error in overestimation or underestimation of the viewing time given the study objective and the characteristics of the stimuli.

**FIGURE 8:**
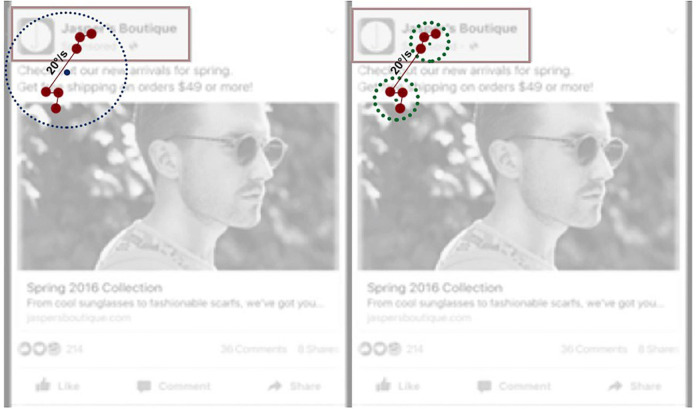
An illustration of the impact of the velocity threshold parameter on the detection and location of fixations on a mock advertisement. On the left side, gaze samples, marked with red dots, are combined into a fixation based on the default threshold value of 30°/s, and the fixation is located outside the AOI Branding. On the right side, the velocity threshold is lowered to <20°/s, resulting in two short fixations with more accurate mean location coordinates.

The error components in TTFF and the ratio of viewers (Percent Seen) were mainly driven by the combination of parameters of merging adjacent fixations and discarding short fixations. For both metrics, the errors are minimized when short fixations are not discarded. The explanation for this finding may be related to the smooth pursuit. When viewers scroll the smartphone screen and attend a dynamic stimulus, their eyes move along with the information presented on the screen. As the event detection algorithm discards the gaze samples with the angular velocity above the threshold value, it is likely that a substantial proportion of short fixations have a duration below 60 ms. Discarding these short fixations, therefore, makes the fixation detection algorithm much more restrictive and conservative, as visualized in Figure 8. As the data reveals, for the metrics TTFF and Percent Seen, keeping the short fixations in the fixation dataset leads to an output that is more similar to the benchmark used in this study, i.e., dwells with the cutoff point of 60 ms.

These results suggest that the algorithm parameters affect the magnitude and directionality of different error components, and that there is a tradeoff between the overestimation and the underestimation of total viewing time. Therefore, the decision related to the improved settings of the fixation detection algorithm must be driven by the study objective and the characteristics of the stimuli. In a setup based on natural viewing behavior of dynamic stimuli on a smartphone screen and focusing on a small AOI such as the branding element, these findings suggest that improved settings are the velocity threshold in the proximity of 10°/s and the function of discarding short fixations disabled. These adjustments to the fixation detection algorithm improve the accuracy of Percent Seen by 19% compared to the output of the Tobii I-VT Fixation Filter, when using the benchmark of Raw60 described above. With this in mind, however, a more practical insight into how well the different Test filters perform can be obtained by investigating the correlations between the output of different Test filters and the benchmark results.

We also find that deciding upon the improved parameter settings depends on which metrics need to be prioritized. For all metrics, it is clear that combinations where the discarding short fixations is disabled perform significantly better than the combinations with the function enabled. The function of merging adjacent fixations has a minor impact on the accuracy of the data output. Disabling the function leads to a slightly more accurate TTFF measures, and enabling it has a slight benefit when focusing on the ratio of viewers, i.e., Percent Seen. When the study objective is related to Percent Seen, velocity thresholds of 5 and 6°/s perform the best, whereas when prioritizing the TTFF metric, thresholds 10 and 11°/s lead to the most accurate results. For TFD, threshold 6°/s yields the results that are most similar to the benchmark of Raw60, but the data output remains fairly accurate also when the velocity threshold is increased up to 10°/s.

While the coefficient of determination values can be considered as indicators of validity of the output of different Test filters, we also found it necessary to assess their reliability. To achieve this, we ran bootstrapped split-half correlations using Spearman correlation values. As Spearman *R* ≥ 0.4 is generally considered acceptable (Fayers and Machin, 2013), we only considered the Test filters with Spearman R equal to or above 0.4 as sufficiently reliable.

In relation to the metrics TFD and Percent Seen, the algorithm parameter settings that displayed the best performance in terms of reliability overlapped to a considerable degree with the findings from the correlation analysis. Considering the reliability of Spearman *R* ≥ 0.4 as the criterion for reliability and combining the results of the correlation and reliability analyses, it could be concluded that for the metric TFD, the algorithm settings with the velocity threshold of 10°/s and the function of discarding short fixations disabled yielded the best results. Also, for the Percent Seen metric, the setting of NoDiscard was preferable, but it is feasible to lower the velocity threshold to 5 or 6°/s. For the metric TTFF the reliability was higher for higher velocity thresholds, but it did not meet the reliability criterion of Spearman *R* ≥ 0.4 for any of the Test filters.

Considering the small size of the AOI and the finding that higher velocity thresholds lead to overly conservative data output and decreased viewer ratio values, it can be inferred that in the case of TTFF reliability, higher thresholds lead to fewer data points and thereby higher reliability values. However, the finding that the TTFF reliability failed to meet the criterion of Spearman *R* ≥ 0.4 regardless of the parameter settings indicates that the metric of TTFF cannot be considered a reliable metric when studying visual attention in the present conditions. For that reason, rather than focusing on TTFF as a millisecond value, it may be worthwhile to focus on the sequential order of fixating on AOIs, i.e., on scan paths instead.

Having demonstrated how different parameter settings of fixation detection algorithms influence different error components, their validity (in the form of coefficient of determination when benchmarked against dwells of 60 ms) and reliability (in the form of Spearman R based on bootstrapped split half tests), we recognize that the choice of the optimized settings of the fixation detection depends greatly on the study objectives and the characteristics of the stimuli. It is recommended by Tobii that the users modify and optimize the settings of the fixation detection algorithm. Here, we provide a framework for how the selection of the improved parameters can be done. As evident from different analyses, the differences in results when comparing the Tobii default Fixation Filter with the Test filters with modified settings are quite substantial. This is exemplified in Table 5. We see from Table 5 that compared to the benchmark Raw60, the default Tobii I-VT Fixation Filter underestimates the viewer ratio (Percent Seen) value by 25%, implying that it fails to capture a quarter of the study participants who viewed the AOI. When using Test filters 2 or 3, the difference in Percent Seen is decreased to 12 and 11%, respectively. From Figure 4 it was clear that threshold values above 10°/s lead to overestimation of the accumulated viewing time. This is likely the reason why the default Tobii I-VT Fixation filter, while missing out on the data of 25% of the viewers, still yields a TFD value that differs from the benchmark Raw60 only by 17%, as compared to the 30 or 23% difference of Test filters 2 and 3, respectively. The TTFF is 60% longer compared to the Raw60 when using the Tobii I-VT Fixation Filter. The difference becomes smaller by using the Test filters 2 or 3 yet is still 44% difference in the best case. Details of these calculations can be found in Table 6.

**TABLE 5:** Comparison of the results of eye-tracking metrics of the benchmark (Raw60), Tobii Fixation Filter (Default) and three Test filters with velocity thresholds 5, 10, and 15°/s (Test filters 1, 2, and 3, respectively).

	Raw60	Default Merge Discard 30°/s	Difference (Raw60 -Default)	Test filter1 NoMerge NoDiscard 5°/sec	Difference (Raw60 – Testfilter1)	Test filter2 NoMerge NoDiscard 10°/s	Difference (Raw60 –Testfilter2)	Test filter2 NoMerge NoDiscard 15(/sec	Difference (Raw60 — Testfilter3)
TFD	0.227	0.188	–0.039 (–17%)	0.123	–0.104 (–46%)	0.16	–0.067 (–30%)	0.174	-0.053 (-23%)
*TTFF*	0.565	0.904	0.339 (60%)	0.874	0.309 (55%)	0.811	0.246 (44%)	0.831	0.266 (47%)
*Percent Seen*	0.495	0.371	–0.124 (–25%)	0.429	–0.066 (–13%)	0.437	–0.058 (–12%)	0.441	–0.054 (–11%)

**TABLE 6:** The definition and calculation of the metrics of interest.

Metric	Definition	Calculated as
Total Fixation Duration (TFD)	The accumulated duration of fixations on a stimulus or an AOI	The sum of the durations of all fixations located on an object or an AOI
Time to First Fixation (TTFF)	The time it takes before a stimulus or AOI is seen	The time that it takes measured from the moment that 50% of the feed post enters the screen until the first fixation lands on that AOI
Percent Seen	The percentage of participants that have at least 1 fixation (= “seen”) on a stimulus or an Area of Interest (AOI)	On a participant level for each AOI, a binary score (SEEN) is assigned (1 or 0, indicating “seen” and “not seen” respectively). This is then aggregated as the percentage of participants that had a minimum of a single fixation on the AOI. This is done by compiling all subjects and taking the percentage of participants who had at least one fixation. For example, if 10 participants were exposed to an AOI and 8 participants saw it for at least one fixation, then %seen would equal 80%, regardless of how long participants viewed the ad for.

## Limitations

This paper is limited to covering a specific AOI size on a mobile phone screen in a natural browsing behavior. While we focused solely on an AOI encompassing the branding area of a mobile feed environment due to the importance of branding in advertising, the findings may not apply to other AOI sizes or other stimulus presentation devices of different sizes and viewing distances. Here, further research must be conducted to answer questions about optimized filters for additional circumstances. Additionally, because we utilized a natural browsing condition in order to observe natural velocities and patterns of eye movements in a vertically scrolling feed environment, conclusions cannot be drawn about the spatial accuracy of eye-tracking on mobile phone screens, since we can only be aware of the observed gaze location rather than the intended gaze location. A follow-up study should be conducted with controlled spatial targets to understand the reliability and validity of spatial accuracy measurements for eye-tracking on smartphone devices, utilizing ROC.

*Our work* focuses on both the validity and reliability of a given measure, although not a comprehensive test that focuses on the desired sample size for each score. Future studies should conduct test–retest and split-half analyses on these and similar data to assess both the general reliability of the selected fixation filter settings, as well as to determine how sample size affects the reliability for small, medium, and large AOIs.

This paper focuses on results using Tobii Pro Glasses 2 50 Hz hardware and Tobii Pro Lab software, and the findings may not apply to other types of eye-tracking hardware and software. Tobii recently released a newer version of eye-tracking glasses hardware, called Tobii Pro Glasses 3, however due to similar hardware specifications and software, we do not expect this hardware difference will have a meaningful impact on the findings presented herein.

Finally, one pertinent limitation of this study is that it does not compare the fixation filter performances to a gold standard measure of gaze movements and fixations. In fact, the study can be seen as a challenge of the long-held industry standard, especially for particular use cases such as eye-tracking on small screens. While this study and its results do not define optimal fixation filters with finality, they imply that further work can be done to improve the accuracy and reliability of eye-tracking measures under different conditions. Here, additional research is needed, and this paper provides one viable path to pursue such research.

## Conclusion

As technology changes and advances, humans shift their behavior to interface with new devices as their primary mode of consumption. With this change in the consumption landscape, researchers often continue to utilize previously used data collection and analysis techniques. While fixations can be a helpful tool in analyzing visual attention in visually cluttered environments, such as advertisements in a social media feeds, the lack of mobile-specific fixations filters renders existing, widely utilized fixation filters ineffective in evaluating advertisements in a vertically scrolling social media feed presented on a smartphone. For improved evaluation of gaze metrics on Branding AOIs for advertisements placed in mobile social media feeds, researchers using Tobii Pro Glasses 2 with Tobii Pro Lab software should use a velocity threshold of 10°/s and disable the function of discarding short fixations, which improves the accuracy of Percent Seen by 19%. Future research should be conducted to validate the spatial accuracy of fixation filters for vertically scrolling content on smartphones. We hope that readers take away from this paper not only an increased knowledge in filter settings for eye-tracking research on smartphones, but also a framework for testing reliability, and we hope further frameworks will be developed and applied for validation testing of datasets in related fields.

## Data Availability Statement

The raw data supporting the conclusions of this article may be made available, pending legal review at the affiliated institutions. Requests to access the datasets should be directed to JT, juliatrabulsi@fb.com.

## Ethics Statement

The patients/participants provided their written informed consent to participate in this study. Written informed consent was obtained from the individual(s) for the publication of any potentially identifiable images or data included in this article.

## Author Contributions

JT and MS conceived and designed the original research. MS was responsible for data collection. SS, KN, and TR completed the data processing and analysis. All authors contributed to writing and editing the manuscript.

## Conflict of Interest

JT is employed at Facebook Inc. KN, MS, and TR are employed at Neurons Inc. The design, analysis, and interpretation of the results of this study have not been affected by these affiliations. The remaining author declares that the research was conducted in the absence of any commercial or financial relationships that could be construed as a potential conflict of interest.

## Publisher’s Note

All claims expressed in this article are solely those of the authors and do not necessarily represent those of their affiliated organizations, or those of the publisher, the editors and the reviewers. Any product that may be evaluated in this article, or claim that may be made by its manufacturer, is not guaranteed or endorsed by the publisher.

## Footnotes

1
https://www.tobii.com/

2
https://www.datatilsynet.dk/generelt-om-databeskyttelse/lovgivning/

3
https://ec.europa.eu/info/law/law-topic/data-protection_en

4
https://www.nmsba.com/buying-neuromarketing/code-of-ethics
