# Supplementary material for: Optimizing Fixation Filters for Eye-Tracking on Small Screens
Source: Front Neurosci. 2021 Nov 8;15:578439. doi: 10.3389/fnins.2021.578439 (PMC8606821; doi:10.3389/fnins.2021.578439)
Supplement: Supplementary file 1 [file Data_Sheet_1.PDF]

Supplementary material for the research article

Optimizing fixation filters for eye-tracking on small screens

By: Julia Trabulsi, Kian Norouzi, Seidi Suurmets, Mike Storm, Thomas Zoëga Ramsøy

Tobii Pro Glasses 2 and Tobii Pro Lab software allows researchers to record and analyze eye movements in naturalistic settings with high resolution and accuracy. In order to detect and extract fixations, the software relies on I-VT fixation algorithm that calculates the angular velocity for each data point and, based on specific parameters, classifies gaze samples as being part of a fixation or a saccade. The problem is, however, that the default settings for the fixation classification function are developed in a stationary study setup where stimuli are displayed on a computer screen that is larger in size and located at a further distance, as compared to a setup where respondents look at the stimuli on a smartphone screen. The authors suggest that the settings of the I-VT fixation classifier need to be adjusted based on the stimuli and the eye tracker used. However, developing the optimized parameters for smartphone-based presentation of dynamic stimuli is not straightforward and requires testing various combinations of different parameter adjustments.

The purpose of this supplementary material is to describe the method and the procedures that were undertaken to determine the optimized settings and criteria for the analysis of eye-tracking data from mobile environments, and more specifically when stimuli are presented on the screen of a smartphone.

To determine the parameters of the gaze filters to be modified, it was decided to run a pilot test where the viewers' selection of gaze is strictly controlled. We could have applied fixation filters with different parameters to existing mobile eye-tracking datasets, but doing so, it would have been impossible to distinguish between gaze samples that were voluntarily selected by the viewer and the noise that resulted from oculomotor processes and the process of recording eye movements.

The controlled study is described in section 1.3. It is followed by section 1.4 that describes the selection of parameters for different fixation filters tested.

However, before the impact of different gaze filter parameters on fixation data can be examined, it was found necessary to test two important aspects:

(1) whether the level of gaze remapping, i.e. whether the gaze is remapped as raw samples or fixation data, has an impact on the data output, and
(2) how the adjustments to the velocity threshold parameter impact eye-tracking metrics related to Areas of Interest (AOIs) of different size.

These two investigations were based on a pre-existing dataset, and are described in the following sections.

1.1 The impact of remapping gaze vs. fixations on the data output

As described in Tobii documentation[1], in order to calculate eye-tracking metrics, gaze samples or fixations need to be remapped from scene recordings to a reference image. Tobii Pro Lab software allows to either remap raw gaze samples and apply fixation filters to the remapped data or to remap fixation data that has already been filtered. As the sampling frequency of Tobii Pro Glasses is 50Hz, the former approach implies that per each second of recording there are 50 samples that need to be located and marked on the reference image. Remapping of fixation data, however, is based on remapping of clusters of gaze samples, requiring significantly fewer man-hours.

From the perspective of developing and testing a high number of fixation filters, it was necessary to determine whether we can remap the raw gaze just once and apply all different filters to the remapped raw gaze data, or whether the fixation remapping needs to be done separately for each fixation filter tested.

In order to investigate whether the level of remapping has an impact on the data output, we used a pre-existing dataset where the eye movement data of 60 respondents (mean age 36.6, st. dev 8.92, 33 females) had been remapped to reference images based on both, raw gaze samples and fixations. The dataset consisted of three types of stimuli displayed on a smartphone screen: Static ads, Organic content, and Video ads. Each of these types included 12 different brands, meaning that participants were exposed to a total of 36 stimuli. The eye tracking metrics on each stimulus were computed based on a total of 11 distinct Areas of Interest (AOIs): Post, BrandLogo, BrandName, Comments, Creative, Likes. An example of the definition of AOIs is presented in figure 1.

[1] Tobii Technology. (2018). Tobii Pro Lab User's Manual, *1.102.4*. Retrieved from https://www.tobiipro.com/siteassets/tobii-pro/user-manuals/Tobii-Pro-Lab-User-Manual/?v=1.102

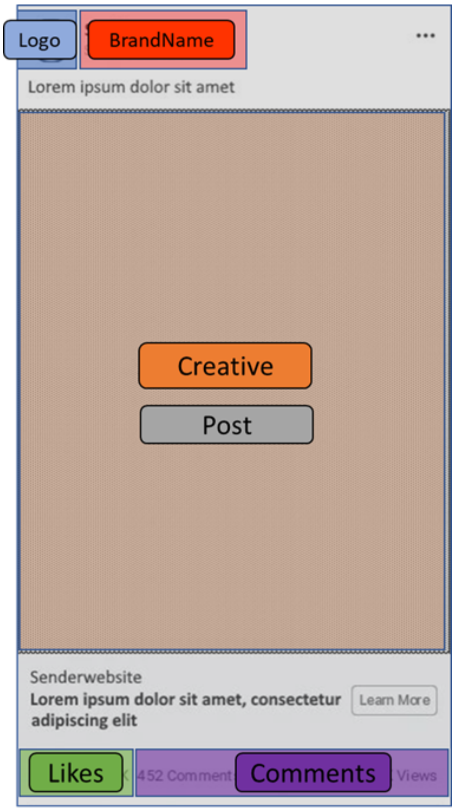

Figure 1: Visualization of AOIs. Note that the AOIs are overlapping - while the AOI 'Post' covers the entire stimulus area, the image or the video in the center of the stimulus area is marked as 'Creative'.

Participant level eye tracking metrics and aggregated metrics were extracted based on (1) remapping of raw gaze and (2) remapping of fixations. In both cases, the default settings of Tobii Fixation Filter were used. To compare the datasets based on different levels of remapping, we used Cronbach's α -- a commonly used measure of internal consistency, where higher values (above .8) are indicative of good reliability of the scale or a measurement instrument.

We compared the datasets based on two eye-tracking metrics that are most commonly used for data analysis: the time respondents spent fixating on the stimulus area (total fixation duration, TFD) and the percentage of stimulus areas seen (%Seen). The datasets were compared both on aggregate and on a participant level, and to further test the sensitivity of the measures, we compared the datasets based on different stimulus types, including only 5 areas of interest. The results of different comparisons are presented in table 1.

Metric	Data type	Stimulus type	AOIs included	Cronbach's α

**Average time spent**	Aggregated	Ad, Organic, VideoAd	All	.974
**Percentage Seen**	Aggregated	Ad, Organic, VideoAd	All	.968
**TFD**	Participant level	Ad, Organic, VideoAd	All	.931
**%Seen**	Participant level	Ad, Organic, VideoAd	All	.916
**TFD**	Participant level	Ad	Creative, BrandLogo, BrandName, Comments, Likes	.908
**TFD**	Participant level	Organic	Creative, BrandLogo, BrandName, Comments, Likes	.987
**TFD**	Participant level	VideoAd	Creative, BrandLogo, BrandName, Comments, Likes	.923
**%Seen**	Participant level	Ad	Creative, BrandLogo, BrandName, Comments, Likes	.885
**%Seen**	Participant level	Organic	Creative, BrandLogo, BrandName, Comments, Likes	.879
**%Seen**	Participant level	VideoAd	Creative, BrandLogo, BrandName, Comments, Likes	.892

Table 1: Comparison of datasets based on the remapping of raw gaze vs. fixation data

As evident from Table 1, in most cases there is excellent reliability (i.e. Cronbach's α >.9) when comparing the two datasets based on different remapping approaches. The measure of internal consistency falls slightly below .9 for % Seen metric when only a single type of stimulus with a limited number of AOIs is included in the analysis. However, considering the limited number of data points and the small size of the AOIs included in the analysis, the Cronbach's α values still indicate very high accuracy.

Considering the high internal consistency, as reflected by high Cronbach's α values when comparing the data on multiple levels of analysis, it can be inferred that the accuracy of the data output is not influenced by whether raw gaze or fixations are remapped. This implies that:

(1) for the study with controlled gaze selection, it is justified to apply different fixation filters to gaze data remapped with raw samples, and
(2) once the optimal parameters for gaze filters have been determined, the remapping can be done based on fixation data output.

1.2 The impact of adjusting the velocity threshold parameter on eye-tracking metrics on AOIs of different size

In order to investigate the degree to which adjustments to the velocity threshold impact eye tracking metrics, six custom gaze filters were created. The only variable adjusted in the filter settings was the velocity threshold parameter, ranging from 10°/s to 15°/s. All other parameters were kept the same as the default settings for Tobii Fixation filter. For the purpose of comparison, also the default Tobii Fixation Filter was included in the analysis.

Figures 2 and 3 visualize how the adjustments to the velocity threshold parameter influence the mean Total Fixation Duration (TFD) for an AOI of a larger size ‘Creative’, versus a smaller AOI ‘Likes’. The first bar on the left side of the graph visualizes mean TFD based on the default settings of Tobii Fixation Filter with the velocity threshold 30°/s. The six bars on the right side of the graph, signified as TFD T10 to TDF T15, visualize the Mean TFD for custom fixation filters with the velocity threshold ranging from 10°/s to 15°/s, respectively.

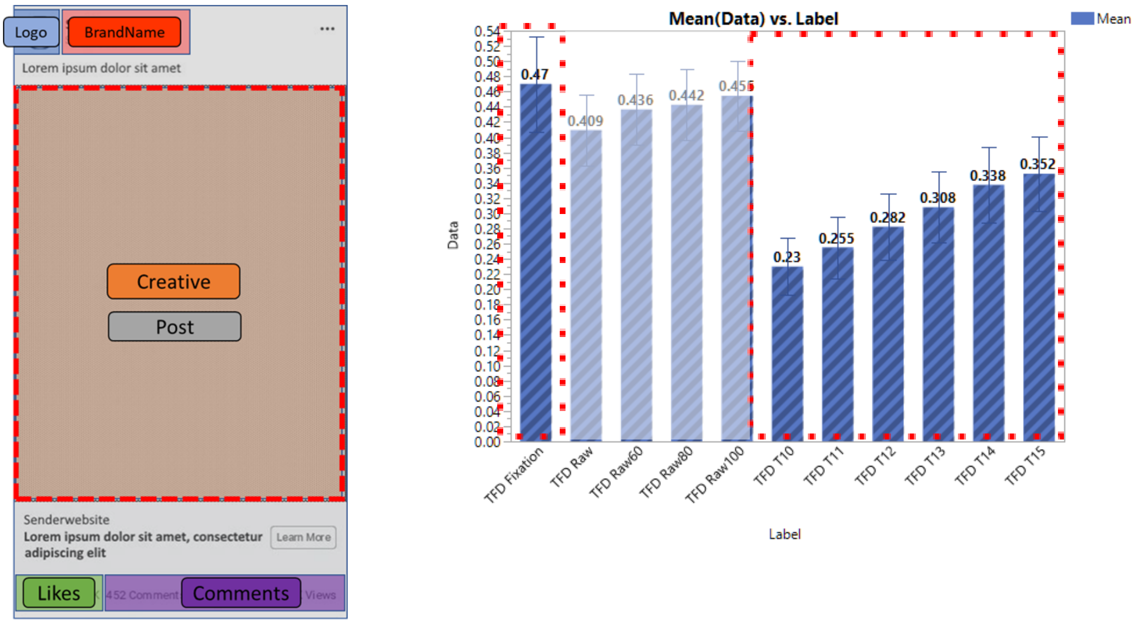

AOI covering a larger area: Creative

Figure 2: Mean Total Fixation Duration (TFD) for the AOI ‘Creative’ and its dependence on the velocity threshold parameter (Default Fixation filter with 30°/s threshold and custom filters with the threshold ranging from 10°/s to 15°/s)

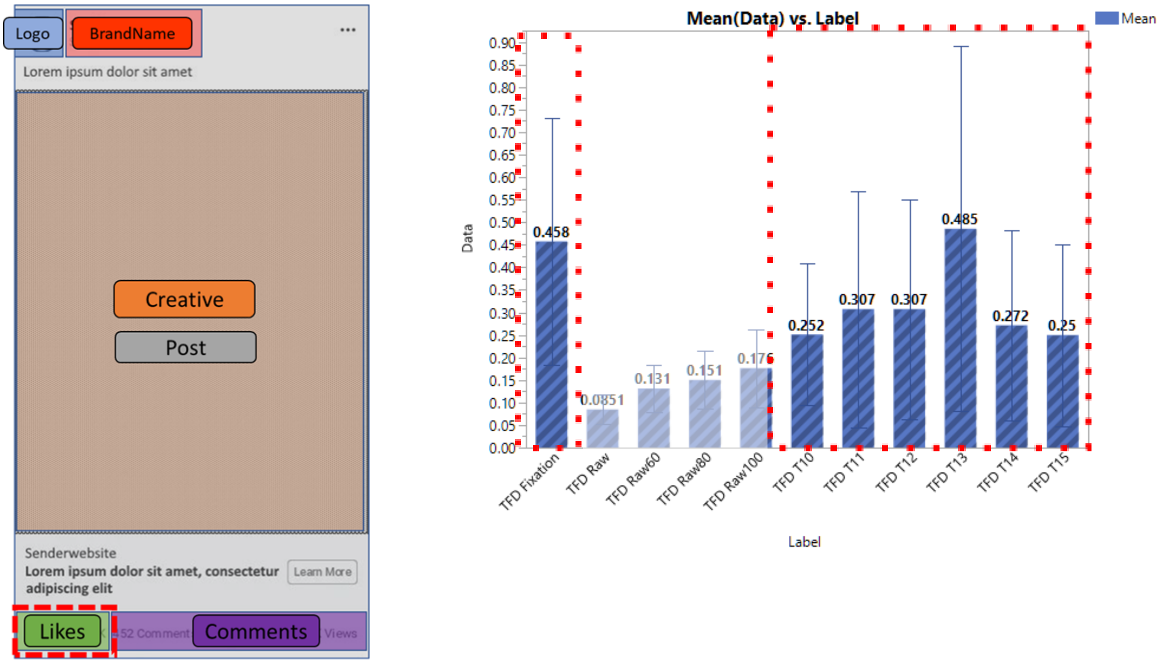

AOI covering a small area:
Likes

Figure 3: Mean Total Fixation Duration (TFD) for the AOI 'Likes' and its dependence on the velocity threshold parameter (Default Fixation filter with 30°/s threshold and custom filters with the threshold ranging from 10°/s to 15°/s)

As the velocity threshold parameter value is lowered, the fixation detection algorithm becomes more sensitive to small movements of the eyes. This means that longer fixations, made up of a longer chain of gaze points, are split into smaller clusters of gaze points. The default setting in Tobii Pro Lab software is to discard fixations with the duration shorter than 60ms. This, in turn, implies that as the velocity threshold is lowered, the fixation data includes fewer gaze points, resulting in decreased mean TFD, as visible in Figure 2.

However, the situation is different when the AOIs are of smaller size, as is the case for AOI 'Likes' in figure 3. Here we see that the velocity threshold 13°/s leads to higher mean TFD than higher thresholds. According to Tobii documentation[2], the Y and X coordinates of a fixation are computed as the arithmetic mean of the coordinates of individual gaze samples. When the velocity threshold is lowered and fixations are made up of a smaller number of gaze samples, the locations of these fixations become more precise. This is visualized in Figure 4, where red dots represent individual gaze points and brown dashed circles visualize how they can be combined as fixations with the center point corresponding to the arithmetic mean of the coordinates.

---

[2] Tobii Technology. (2018). Tobii Pro Lab User's Manual, *1.102.4*. Retrieved from https://www.tobiipro.com/siteassets/tobii-pro/user-manuals/Tobii-Pro-Lab-User-Manual/?v=1.102

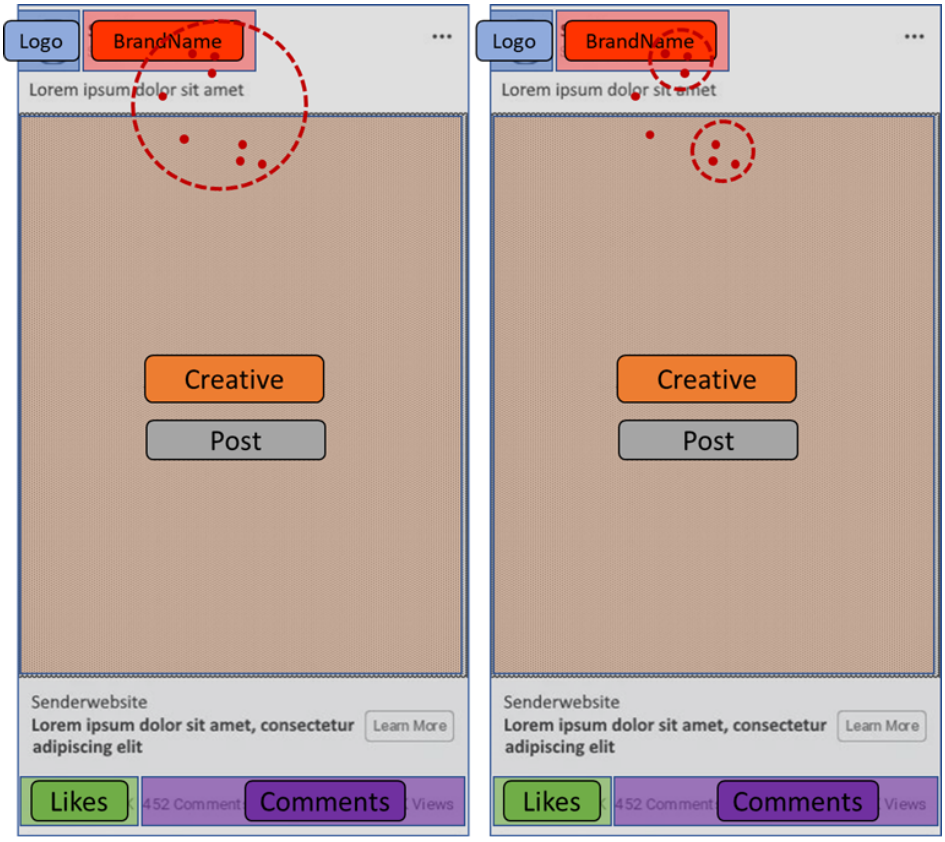

Figure 4: The impact of the velocity threshold parameter on the detection of fixations. Gaze samples, marked with red dots, are combined into fixations, based on a higher (left) versus a lower threshold value (right).

Figure 4 visualizes the impact that the fixation algorithm parameters can have on the accuracy of the eye movement data. The liberal velocity threshold visualized on the left side of figure 4 does not provide sufficient level of detail and combines all gaze points into a single fixation, locating it on the AOI ‘Creative’ (even though a small cluster of gaze points landed on the AOI ‘Likes’).

On the right side of the figure the fixation filter with a lower velocity threshold combines the gaze points into two short fixations. In this case, the gaze points landing on the AOI ‘Likes’ constitute a separate fixation and its parameters are included in the computation of eye-tracking metrics for that specific AOI.

When the fixation filter settings are set more conservative, there is an increased amount of gaze points that are classified as not belonging to a fixation. How these gaze points are handled depends on other parameters, such as merging adjacent fixations and discarding short fixations.

The default setting for Tobii Fixation filter is to merge the adjacent fixations, that occur at less 75ms and 0.5° apart, and to discard fixations that last less than 60 ms. As the individual AOIs displayed on a smartphone screen are relatively small in size, merging adjacent fixations may decrease the accuracy of the data. It is also possible that discarding short fixations leads to a loss of meaningful gaze data. On the other hand, merging adjacent fixations and discarding short fixations may effectively reduce noise and improve the data quality. The analysis of how to handle the gaze points that do not belong to any fixations, with regards to whether they represent noise or meaningful eye movements, is only possible when the viewers' intent during the exposure to the stimuli is known.

1.3 Pilot study based on controlled gaze selection

As previously described, in order to be able to distinguish between gaze samples that are voluntarily selected by the viewer and noise that should be eliminated from the dataset, it was found necessary to conduct a study where viewers' gaze selection would be controlled.

1.3.1 Data acquisition

Tobii Pro Glasses (50/100Hz) were used for data collection and Tobii Pro Lab software was used for recording and pre-processing the data. Stimuli were presented on Samsung Galaxy S7 smartphone with 5.1-inch screen.

The study took place in Neurons Inc offices, in a quiet room with appropriate lighting conditions. The participants were seated at a table and held the smartphone in their hand throughout the session.

1.3.2 Participants

The study was conducted with 13 respondents (mean age 26.7, st. dev 4.78, 3 females), with normal or corrected-to-normal vision and no history of neurological or psychiatric disease. All respondents were employees of Neurons Inc and participated in the study during their office hours.

1.3.3 Procedure

The session started with the technician mounting the Tobii Pro Glasses on the participant's head, making sure that the device stays in place throughout the recording. As the first step, the eye-tracker was individually calibrated by participants focusing their gaze on the center of the target on the Calibration Card. The accuracy of the recording was then tested via live view function, where participants were instructed to look at static target objects displayed on the smartphone screen.

After ensuring accurate calibration, the study session comprising of five different viewing tasks was started.

The participants were instructed to maintain the optimal viewing posture and refrain from moving the phone during the viewing tasks. All instructions related to the viewing tasks were presented in written form on the smartphone screen.

After the viewing tasks were completed, the accuracy of the gaze recording was again tested based on static target objects. The participants were then thanked for their time and could leave the testing area.

1.3.4 Stimuli

The study comprised of 5 different viewing conditions where respondents were instructed to look at the target objects displayed on the smartphone screen. In all conditions the target object was displayed as a light gray cross with a circle displayed around it, displayed on a dark gray background.

The design of the target object was based on the following considerations:

> (1) the cross with the circle around it allows the viewers to focus on the center of the target, but enables objective gaze remapping, when deciding whether gaze points landed on the target or not;
>
> (2) the colors of the background and the target object should have a good contrast balance but should not excessively tire the eyes.

In order to test investigate eye movements in conditions where viewers' gaze follows dynamic stimuli, also conditions with moving targets were included. To correspond to different scrolling speeds, it was chosen to test three speeds during which the target moved from the bottom to the top of the screen: 3 seconds, 1 second and 500 milliseconds. All instructions and all 5 viewing tasks, including different speed conditions, were presented as a single video. The description of the stimuli and the instructions are presented in table 2.

Description of stimuli				
One static target changing its location<br>10 targets in total<br>Each displayed for 5 sec at a time	One static target changing its location<br>30 targets in total<br>Each displayed for 1 sec at a time	Two targets displayed at the same time<br>Lasting 30 sec in total	A target moving from the bottom of the screen to the top of the screen.<br>Displayed at three different speeds: (1) 3 sec., (2) 1 sec. (3) 500 ms.<br>10 targets at each speed	Two targets displayed at the same time, moving from the bottom of the screen to the top of the screen<br>Displayed at three different speeds: (1) 3 sec., (2) 1 sec. (3) 500 ms.<br>20 targets at each speed
**Appearance of stimuli**				
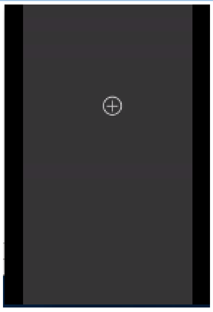	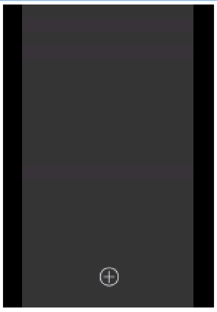	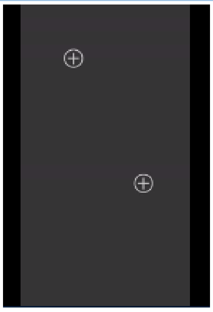	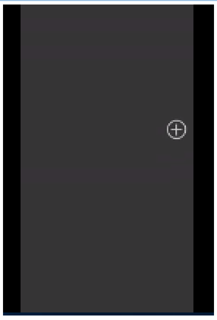	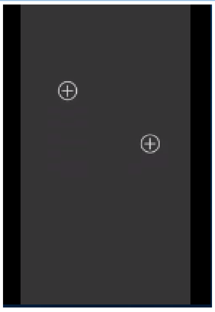
**Task instructions**				
For the next minute, crosses will be displayed on the screen. Please look at the crosses at all times (focus on the middle of the cross).	For the next task, crosses will be displayed on the screen. Please look at the crosses at all times.	For the next task, TWO crosses will be displayed on the screen at the same time. Please, look at one cross at a time, swapping your gaze between the two crosses. You can do it at your own pace.	For the next task, crosses will appear from the bottom of the screen moving upwards. Please follow the crosses, looking at them at all times.	For the next task, crosses will appear from the bottom of the screen moving upwards. TWO crosses will be displayed at the same time. Please, look at one cross at a time, swapping your gaze between the two crosses. You can do it at your own pace.

Table 2: Study design for controlled gaze selection

1.4 Variables

The focus of the data analysis is based on classifying gaze points either as ‘hits’, i.e. landing on the target object, or ‘noise’, i.e. landing on other areas of the visual stimulus. In order to do so, raw gaze points are mapped on reference images corresponding to each of the conditions. Different fixation filters are then applied to the remapped gaze data.

The main objective of the data analysis is to estimate the degree to which each of the fixation filters tested correctly classify the ‘hits’ on targets as fixations, as opposed to how much ‘noise’ is included in the dataset.

1.4.1 Determining the parameters for fixation filters to be tested

Based on the analysis of different velocity thresholds on eye-tracking metrics, presented in section 1.2, it was decided to test the modification of the following parameters:

- Discarding short fixations (default setting: fixations below 60ms are discarded) – if the viewer's gaze follows a dynamic stimulus presented on a smartphone screen, the accuracy of the data input may improve if short fixations with the duration lower than 60ms are included in the dataset
- Merging adjacent fixations (default setting: fixations within 0.5° proximity are merged) – because a smartphone occupies only a small area of the viewer's visual field, merging adjacent fixations may lead to incorrect estimation of fixations
- Velocity threshold (default setting: consecutive gaze points with the angular velocity up to 30°/sec are regarded as belonging to the same fixation)- the velocity of eye movements is influenced by the size and the proximity of the stimulus and lowering the velocity threshold is likely to result in more detailed data (i.e. larger number of short fixations)

Using Tobii Pro Lab interactive software which allows investigating how the alteration of different parameter settings influences the classification of fixations, and comparing the data output to the scene recordings, it was found that velocity thresholds in the proximity of 10 °/sec provide the closest reflection of the actual eye movements. It was found that while 5°/sec threshold sliced fixation data to overly small clusters of gaze points, the threshold of 15 °/sec merged gaze movements over several AOIs into long ambiguous fixations. Accordingly, these two values were decided to be taken as the two border conditions, implying that all discrete velocity values ranging from 5 to 15 °/sec would be tested as fixation filter parameters.

Figure 5 visualizes the fixation data output when the velocity threshold is set to 5°/sec (left), 10°/sec (middle) and 15 °/sec (right). Each horizontal bar signifies a chain of gaze points that are classified as a fixation. The pink lines at the bottom of the graph indicate the velocity values of the gaze points.

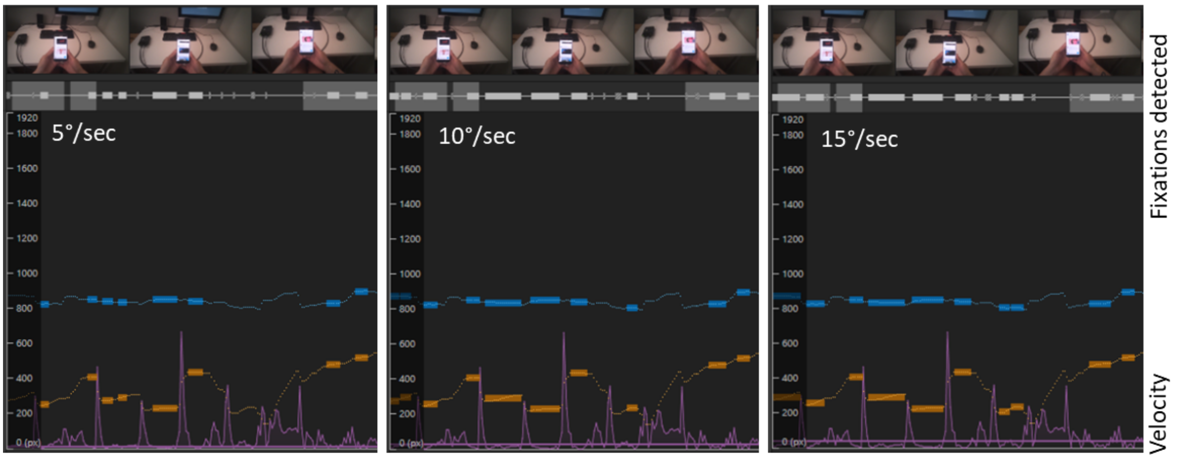

Figure 5: Fixation data output as the velocity threshold is modified. Each horizontal bar signifies a chain of gaze points that are classified as a fixation.

From figure 5 it is visible that while the velocity threshold 5 °/sec is overly strict in terms of classifying gaze points as fixations, the threshold 15°/sec is too liberal and likely to lead to inaccurate data. The optimal parameter is therefore likely to reside somewhere in between these two border conditions tested.

These visualizations in figure 5, however, still portray the default settings where adjacent fixations are merged and short fixations are discarded. Different adjustments to these three parameters lead to different outputs of the fixation data, and accordingly, it was decided to test 44 different gaze filters: Discarding short fixations (2) x Merging adjacent fixations (2) x Velocity threshold (11). In addition to the 44 test filters, it was decided to include a total of 12 filters with velocity thresholds 20°/sec, 25°/sec and 30°/sec. It was done in order to investigate how increased velocity threshold impacts the data output, and to benchmark different filters against the default Tobii Fixation Filter (i.e. threshold 30°/sec, merging adjacent fixations enabled, discarding short fixations enabled). The 44 test filters together with the additional 12 filters with adjusted parameters are presented in Table 3.

Merging adjacent fixations	Discarding short fixations	Velocity Threshold													
		**5**	**6**	**7**	**8**	**9**	**10**	**11**	**12**	**13**	**14**	**15**	**20**	**25**	**30**
**Enabled**	**Enabled**	F1	F2	F3	F4	F5	F6	F7	F8	F9	F10	F11	F45	F46	F47
Disabled	**Enabled**	F12	F13	F14	F15	F16	F17	F18	F19	F20	F21	F22	F48	F49	F50
**Enabled**	Disabled	F23	F24	F25	F26	F27	F28	F29	F30	F31	F32	F33	F51	F52	F53
Disabled	Disabled	F34	F35	F36	F37	F38	F39	F40	F41	F42	F43	F44	F54	F55	F56

Table 3: Customized fixation filters tested to determine the optimal parameters for smartphone viewing condition. The fixation filter labeled as F47 represents the Tobii default Fixation Filter.

As the next step, each of the filters presented in Table 3 are tested and compared to other filters with regards to the proportion of gaze points that are correctly classified as fixations landing on the targets, versus how much 'noise' is included in the dataset.

1.4.2 Data pre-processing

All gaze was remapped from scene recordings to reference images, where the main areas of interest (AOIs) included the 'Target' (regarded as Hit) and 'AnywhereElse' and 'BetweenCrosses' (regarded as Noise). Also overlapping AOIs (e.g. 'LeftCross', 'RightCross') were included for potential additional tests. However, the current analysis is based solely on the distinction between Hits (i.e the gaze point is recorded on the Target) and Noise (i.e. the gaze point is recorded elsewhere on the screen). An example of AOI reference image is shown in figure 6.

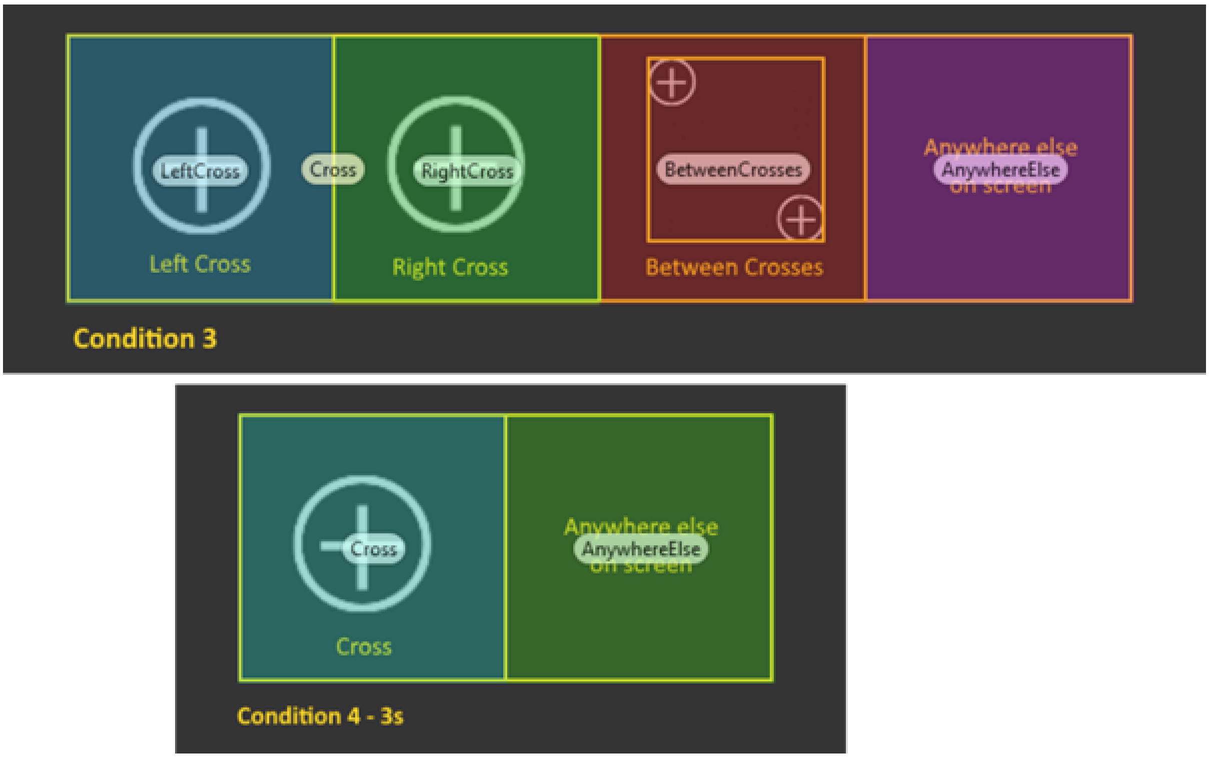

Figure 6: Reference images used for gaze remapping

To remap and analyze the data, Tobii software visualizes eye movements by projecting the gaze points on the video recordings. The size of these projected gaze markers can be adjusted, and the larger the gaze marker, the more liberal (and less accurate) the gaze remapping can be considered to

be, leading to a generally higher sensitivity and lower specificity. Thus, by larger gaze markers, one can expect to both have more True Positives and False Positives.

Gaze mapping was performed based on the projection with the 25% gaze marker size, that was slightly bigger than the size of the targets displayed on the smartphone screen.

If the gaze projection was located directly on the target or its borders overlapped with the borders of the target, it was considered a 'Hit'. If the gaze projection had no overlap with the borders of the target it was marked as 'Noise'.

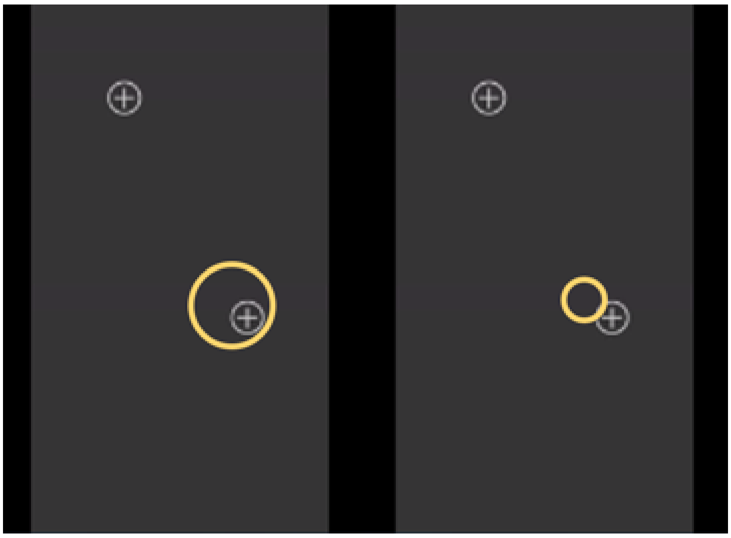

Figure 7: Different sizes of gaze projection. All data was marked based on the projection size of 25% (left), and the gaze sample was considered a 'Hit' when the border of the projection overlapped with the border of the target. For comparison, a more conservative projection size of about 10% is visualized on the right. As this shows, we have chosen to be more inclusive in our markings, as we find that gaze proximate to a target can still lead to processing of that target.

Gaze remapping was based on raw gaze samples. As the frequency of the eye-tracker is 50Hz, a new raw sample is recorded at every 20ms. After all raw gaze was remapped, a total of 56 different fixation detection algorithms were applied to the data.

1.4.3 The proportion of noise recorded during different viewing tasks

To assess the accuracy of the eye-tracking data, as well as the proportion of noise recorded in different viewing conditions, the total fixation duration (TFD) values of Target were compared to the TFD values classified as Noise. Figure 8 visualizes the TFD on Target (green) vs. TFD on Noise (red) in three different viewing conditions:

1. Switching between two static crosses
2. Following a single target, moving at 3 sec. speed
3. Following a single target, moving at 1 sec. speed

The Y-axis in figure 4 denotes the value of TFD and the X-axis visualizes different velocity thresholds. Green and red bars indicate the mean TFD values for Target and Noise, respectively, and the whiskers signify 95% confidence intervals. Each bar represents a different fixation filter tested, whereas the data is divided into 5 sections, depending on the parameters of 'Merging adjacent fixations' and 'Discarding short fixations.' The section on the far right shows the TFD values based on raw gaze samples.

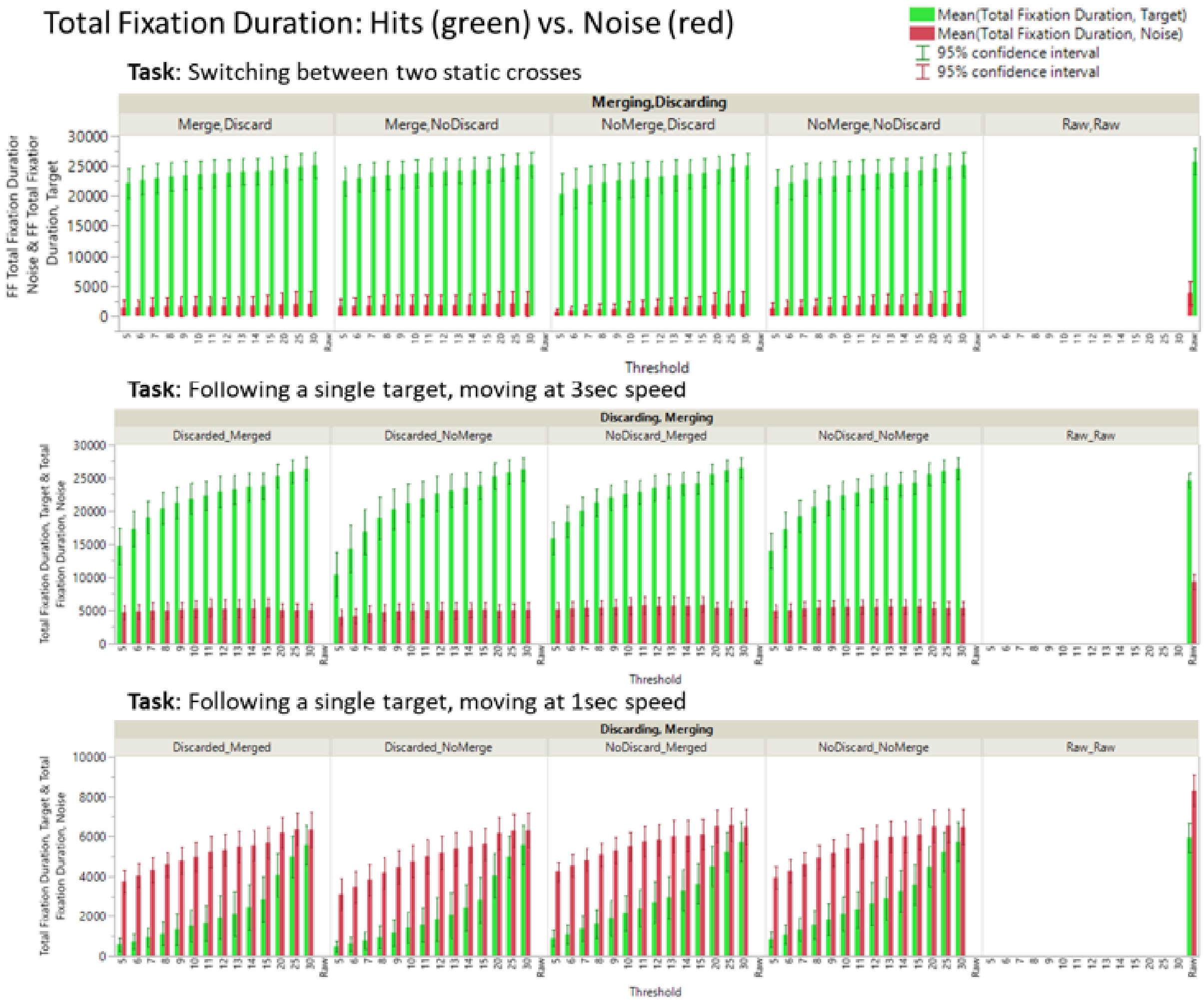

Figure 8: The proportion of Hits (green) relative to the proportion of Noise (red) in three viewing conditions, using both different filters for merging and discarding, and different fixation thresholds. The full description can be found in the text on the previous page.

From figure 8 it is evident that very little noise is recorded when the participants' task is to swap between two static crosses. This implies that the eye movement recordings are very accurate and that participants perform very well when the gaze selection is based on static targets. However, when participants are instructed to follow a moving target, there is a lot more noise. This means that smooth pursuit, in general, introduces a lot of noise in the data, and participants appear to face difficulties when focusing their gaze on a moving target. It is also evident that as the speed of the moving target is increased, the proportion of noise in the data increases dramatically.

Considering the natural viewing condition where participants scroll a feed on a smartphone, it is essential that the parameters of the fixation filter are optimized to capture eye movements during smooth pursuit as accurately as possible. For that reason, the analysis focuses primarily on the gaze data in viewing conditions where the target is moving.

1.4.4 Data analysis: following a moving target at 3sec speed

1.4.4.1 Computation of True Positives, True Negatives, False Positives and False Negatives

As the first step, eye movement data was analyzed based on the task where participants were instructed to follow a moving target on the screen at 3 sec. speed. It can be argued that this condition bears the closest resemblance to the natural smartphone viewing condition where viewers scroll through the feed.

Inspired by classical psychometric approaches including the use of Receiver Operating Characteristics (ROC) curve [3] (i.e. a diagnostic test where the rate of True Positives is plotted against the False Positives rate), we computed True Positives, False Positives, True Negatives and False Negatives for TDF values for different fixation filters. In order to do so, the TFD values based on raw gaze were regarded as the benchmark. Figure 9 presents the computation of these values.

	Actual gaze	
Fixation Filter (FF)	Target	Noise
Target (FF)	**True positives** - classifying target as target (Nr. 1, 2)<br>If ((TFD(**Raw**)Target – TFD(**FF**)Target) < 0 then TFD(**Raw**)Target<br>else = TFD(FF) Target	**False positives** – classifying noise as target (Nr. 5, 6)<br>If (TFD(**FF**)Target – TFD(**Raw**)Target) < 0 then 0<br>else = TFD(**FF**) Target – TFD (**Raw**) Target
Noise (FF)	**False negatives** – classifying target as noise (Nr. 7, 8)<br>If ((TFD(**Raw**) Target – TFD(**FF**) Target) < 0 then 0<br>else =TFD(**Raw**)Target– TFD (**FF**)Target	**True negatives** – classifying noise as noise (Nr. 3, 4)<br>If ((TFD(**Raw**)Noise – TFD(**FF**)Noise) < 0 then TFD(**Raw**)Noise<br>else = TFD(FF) Noise

[3] Thomas A. Lasko, Jui G. Bhagwat, Kelly H. Zou, Lucila Ohno-Machado, The use of receiver operating characteristic curves in biomedical informatics, Journal of Biomedical Informatics, Volume 38, Issue 5, 2005, Pages 404-415

Figure 9: Computation of True Positives, False Positives, True Negatives and False Negatives based on TFD values.

The computation of these values is further described below:

- **True Positives** (classifying target as target) were regarded as the total TFD duration on the target based on the fixation filter, but only when it was smaller than the TFD based on Raw gaze. If TFD based on the fixation filter exceeded the TFD based on raw data, the latter value was regarded as the True Positives.
- **True Negatives** (classifying noise as noise) were regarded as the total TFD duration classified as noise based on the fixation filter, but only when it was smaller than the TFD based on Raw gaze. If TFD based on the fixation filter exceeded the TFD based on raw data, the latter value was regarded as the True Negatives.
- **False Positives** (classifying noise as target) were regarded as the difference between the TFD on target based on the fixation filter and the TFD based on raw data. If TFD based on the raw data exceeded the TFD based on the fixation filter, the value of False Positives was set as zero.
- **False Negatives** (classifying target as noise) were regarded as the difference between the TFD on target based on raw data and the TFD based on the fixation filter. If TFD based on the fixation filter exceeded the TFD based on the raw data, the value of False Negatives was set as zero.

As the next step, the values of True Positives, False Positives, True Negatives and False Negatives were normalized. This was done by dividing the values with the aggregated gaze duration based on raw data (i.e. the sum of TDF based on raw gaze data on target and noise). To obtain percentages, the values were multiplied by hundred. This can be expressed as:

$$Normalized\ (x) = x/(TFD(Raw)Target + TFD(Raw)Noise) * 100$$

where x signifies the specific value of True Positives, False Positives, True Negatives or False Negatives.

1.4.4.2 False Positives

An optimal gaze filter can be regarded as *one that maximizes the proportion of correct classifications and minimizes the proportion of False classifications*. However, when comparing TFD values based on fixation filter output and raw gaze samples, it is natural to have a certain proportion of False Negatives in the data. TFD based on raw gaze should generally be longer than the TFD based on

fixation data, because raw gaze data also indiscriminately contains all hits on a target, including instances where the eyes are on the target but would not formally count as a fixation, thereby representing a slight overestimation of the viewing time. This could include saccades or when the target is midway between point A and B.

The situation is different when the data contains False Positives. This is driven by instances where the fixation filter merges together a number of gaze points that have actually landed outside the area of interest. False Positives can be regarded as indicators of inaccuracy and unreliability of the fixation filter, and should be sought to be as low as possible.

Figure 10 visualizes the normalized number of False Positives ("norm FP") for each of the 56 fixation filters tested. The filters are grouped based on the parameters of discarding and merging fixations and X-axis presents different velocity thresholds.

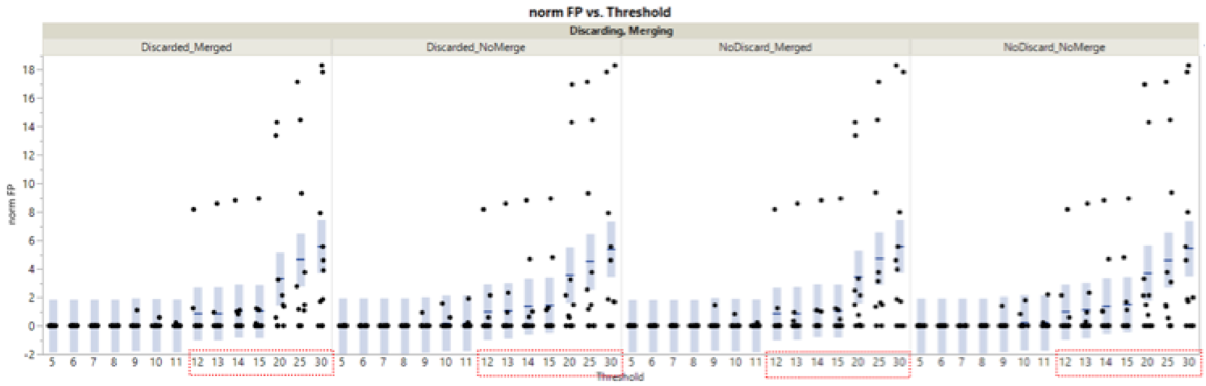

Figure 10: Normalized value of False Positives (FP), as a function of velocity threshold and the parameters of merging/ discarding fixations. Each dot represents a different participant, the blue line denotes the mean value, and the gray areas signify 95% confidence intervals. Red insets denote thresholds where False Positives show a significant increase.

It is evident from figure 10 that velocity thresholds starting from 12°/sec. introduce an increasing number of False Positives to the data. In other words, if the velocity threshold is 12°/sec. or higher, we can see that the fixation filter merges together gaze points that landed outside the target, resulting in a situation where the TFD based on a fixation filter on the target is higher than the TFD based on raw gaze samples.

1.4.4.3 True Positives, True Negatives and the indicators combined

To optimize the proportion of correct classifications, it is important to look into both True Positives and the True Negatives. It can be argued, however, that given the stimulus characteristics (i.e. a small

target on the screen of a smartphone), the classification of True Positives is significantly more important than the classification of True Negatives.

Figure 11 visualizes the normalized number of True Positives (blue) and True Negatives (red) for each of the 56 fixation filters tested.

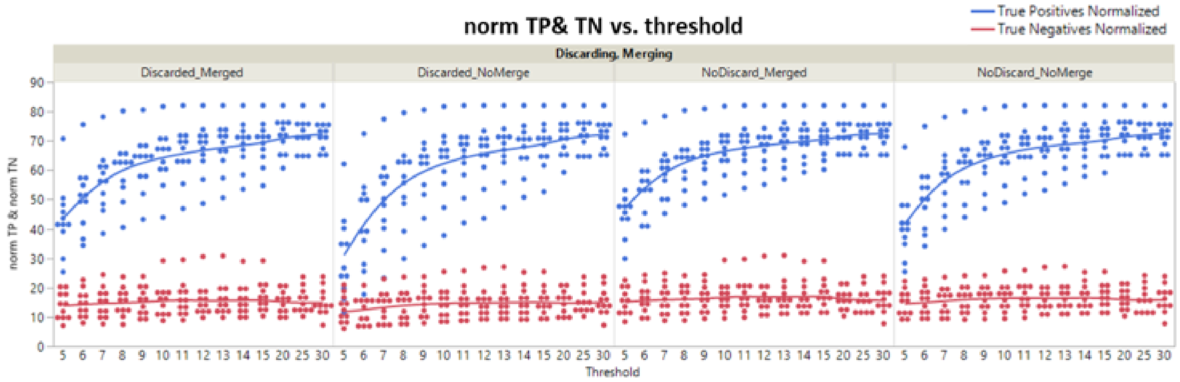

Figure 11: Normalized value of True Positives (TP, blue) and True Negatives (TN, red), as functions of velocity threshold and the parameters of merging adjacent fixations and discarding short fixations.

It is evident that adjustments to the velocity threshold do not have much impact on the True Negatives, as the red line stays on the same level across all velocity thresholds. However, the positive increase in the blue line indicates that the proportion of True Positives increases as the threshold is increased, and that this increase is most pronounced for the “lower” filters, and with diminishing change for the “higher” filters.

When combining all indicators, as visible in figure 12, it is evident that an increase in velocity threshold leads to a decrease in False Negatives (orange line), whereas the increase in True Positives (blue line) is accompanied by an increase in False Positives (green line) for the “higher” filters.

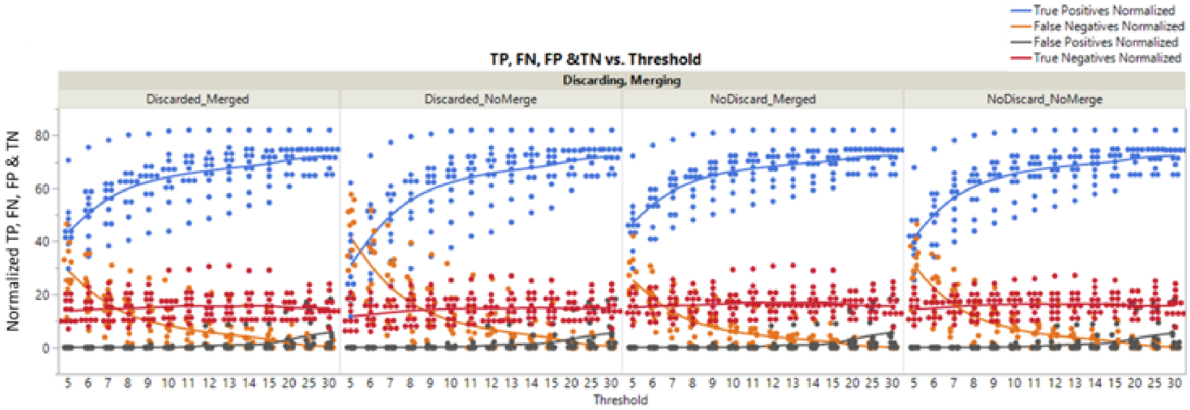

Figure 12: True Positives, True Negatives, False Positives and False Positives combined

Having established that True Positives (TP) and False Positives (FP) are most crucial when determining the optimal fixation filter parameters, we ran a least squares regression based on REML with normalized TP and FP as dependent variables and Discarding /Merging, Threshold and the interaction between the two as model predictors.

The results revealed that for True Positives, the main effects of Discarding/Merging and Threshold are both significant model predictors. For False Positives, only the velocity threshold is a significant model predictor.

DV: **Norm. True Positives**

Discarding/Merging: $F(3, 560)=6.60$, $p<.001$

Threshold: $F(13, 560)=47.04$, $p<.001$

Discarding/Merging* Threshold: $F(39, 560)=.58$, $p<.98$

DV: **Norm. False Positives**

Discarding/Merging: $F(3, 560)=0$, $p=1$

Threshold: $F(13, 560)=14.89$, $p<.001$

Discarding/Merging* Threshold: $F(39, 560)=.01$, $p=1$

1.4.5 Data analysis: following a moving target at 1sec speed

The task of following a target moving at 1sec speed appeared to be rather challenging for the participants, as is evident from figure 13, where the duration of gaze landing on the targets is shorter than the duration of gaze classified as noise.

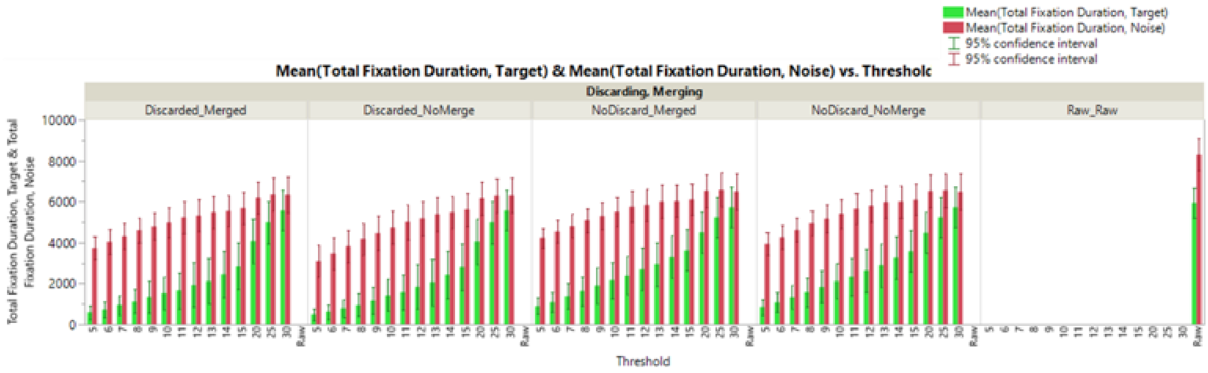

Figure 13: The proportion of 'Hits' (green) relative to the proportion of Noise (red): The whiskers signify 95% confidence intervals.

When analyzing the data for the task ‘Following a moving target at 1 sec. speed’, the steps related to computing True Positives, True Negatives, False Positives and False Negatives were identical to those presented in section 1.4.4.1.

False Positives, True Positives and all indicators combined

The data analysis reveals that when the target is moving at a faster pace, i.e. at 1sec speed, it is less likely to have False Positives in the dataset. It may be due to the reason that overall, there are more gaze points classified as ‘Noise’ than ’Hits’. As seen in figure 14, it is visible that False Positives only occur when the velocity threshold is increased to 25°/sec or higher.

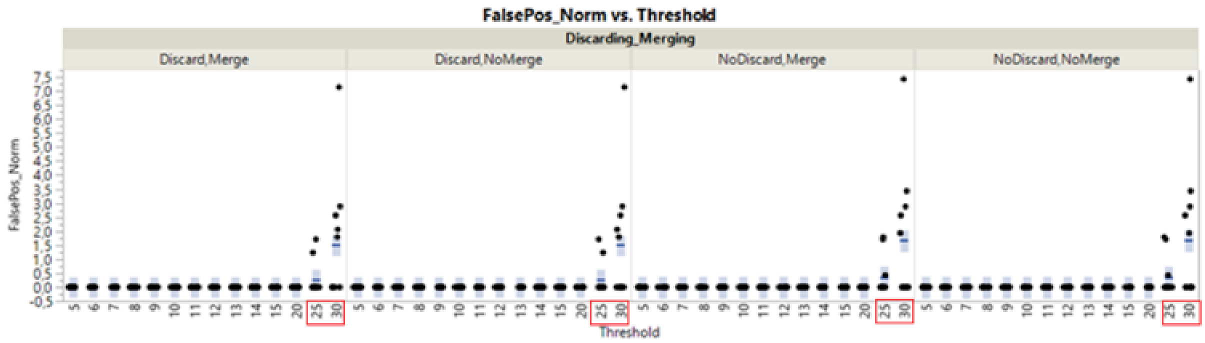

Figure 14: Normalized value of False Positives (FP), as a function of velocity threshold and the parameters of discarding/merging fixations, where grey areas signify 95% CI. Dots denote individual data, blue line indicates mean value, gray area denotes 95% CI. Red squares indicate where the number of False Positives occur.

When True Positives and True Negatives are mapped against the parameters of discarding/ merging and different velocity thresholds, as visible below, it is evident that higher velocity thresholds lead to an increase in both, the proportion of True Positives, as well as True Negatives.

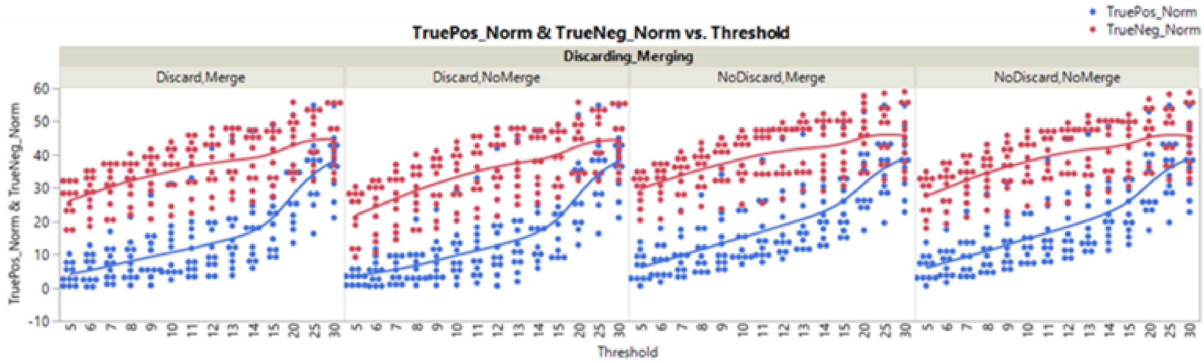

Figure 15: Normalized value of True Positives (blue) and True Negatives (red), as functions of velocity threshold and the parameters of merging/ discarding fixations.

Figure 16 visualizes the normalized proportion of True Positives, True Negatives, False Positives and False Negatives as the velocity threshold and the parameters of discarding and merging are changed. Here the proportion of False Positives remains very low up to the threshold 25.

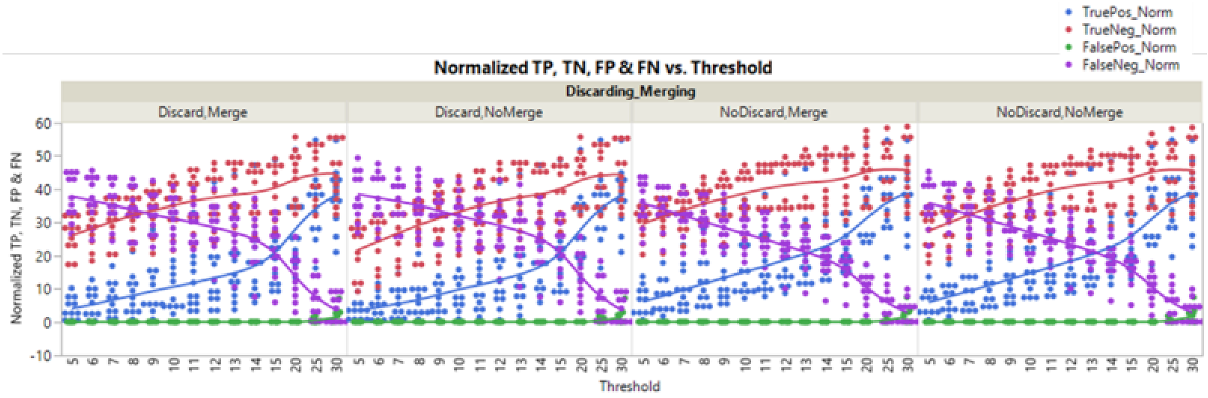

Figure 16: True Positives, True Negatives, False Positives and False Positives combined

Relying on the proportion of True Positives (TP) and False Positives (FP) as the main indicators for determining the optimal fixation filter parameters, we ran a least squares regression based on REML with normalized TP and FP as dependent variables and Discarding /Merging, Threshold and the interaction between the two as model predictors.

The results revealed that for both True Positives and True Negatives, only the velocity threshold is a significant model predictor. The main effects of Discarding/Merging and the interaction between the threshold and Discarding/Merging are not significant.

DV: **Norm. True Positives**

Discarding/Merging: $F(3, 560)=0.26$, $p=.85$

Threshold: $F(13, 560)=61.62$, $p<.001$

Discarding/Merging* Threshold: $F(39, 560)=.12$, $p=1$

DV: **Norm. False Positives**

Discarding/Merging: $F(3, 560)=0$, $p=1$

Threshold: $F(13, 560)=19.57$, $p<.001$

Discarding/Merging* Threshold: $F(39, 560)=.002$, $p=1$

1.4.6 Data analysis: Switching between two static targets

We finally turn to the case of switching between two static targets. This analysis will only be run as an additional, explorative analysis to demonstrate whether the filters identified through the previous sections are viable for this test condition.

As a reminder, this condition saw the highest level of hits vs noise (figure 17):

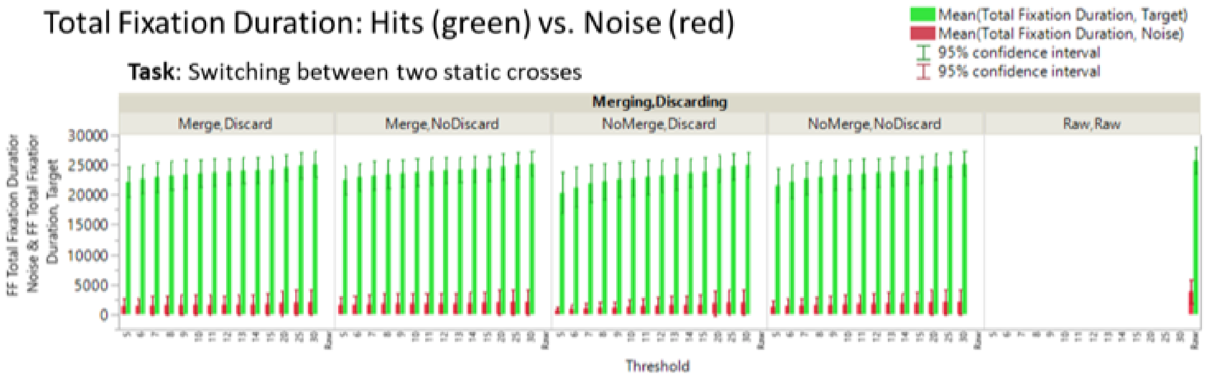

Figure 17: The proportion of "hits" (green) relative to "noise" (red). Bars denote mean values and whiskers denote 95% confidence interval.

As these results clearly show, the task of switching between two static targets produces a high degree of hits and a low degree of noise. The ratio even seems better than the raw gaze data, suggesting that raw gaze produces more noise.

Moving over to False Positives, we can see that at the level of filter 12 (for merge and no discard) there is an increase in False Positives. For other conditions, thresholds up to 15 seem to produce no False Positives rates. This is visualized in figure 18:

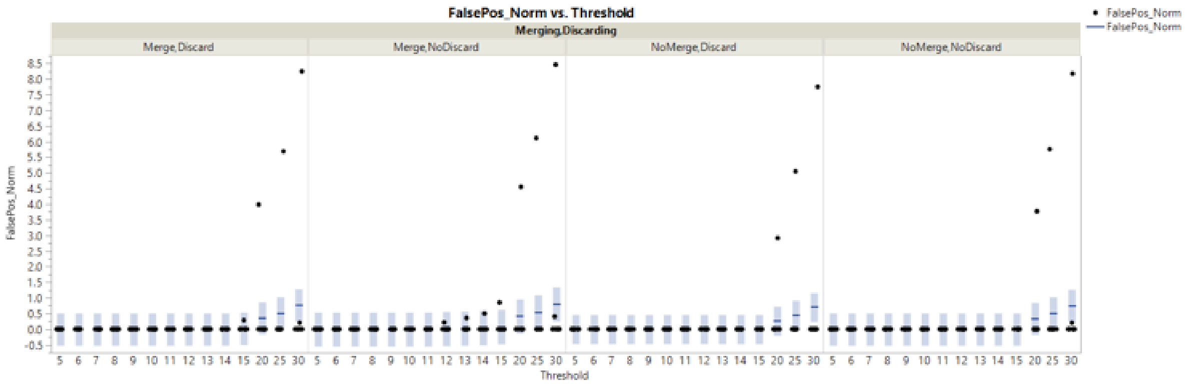

Figure 18: Normalized value of False Positives (FP), as a function of velocity threshold and the parameters of discarding/merging fixations. Dots denote individual data, blue line indicates mean value, gray area denotes 95% CI. Red squares indicate where the number of False Positives occur.

Mapping True Positives and True Negatives against discarding/merging at different threshold, we see that there is a steady incline in scores for True Positives (blue) but that True Negatives remain largely

unaffected. A statistical model further demonstrates this as there is a significant relationship between velocity threshold and True Positives ($F=3.54$, $p<0.0001$) and that even for True Negatives there is a significant difference between the thresholds ($F=3.74$, $p<0.0001$).

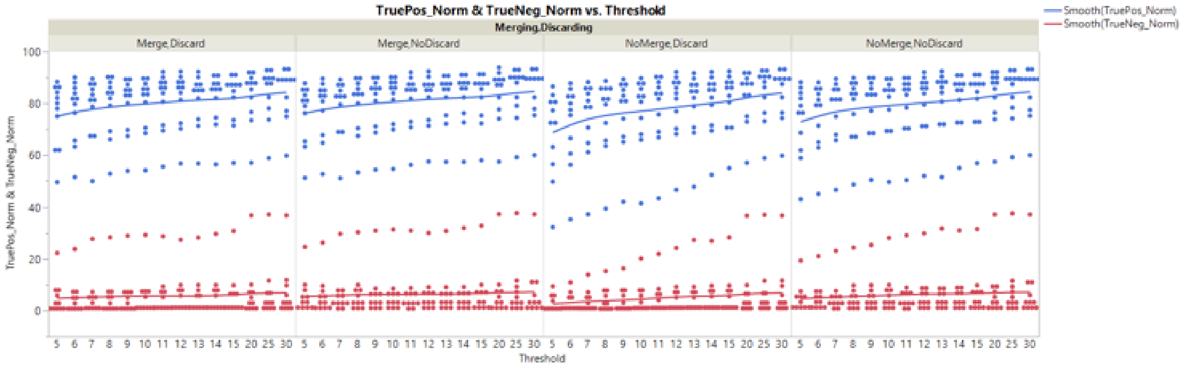

Figure 19: Normalized value of True Positives (TP) and True Negatives (TN), as functions of velocity threshold and the parameters of merging/ discarding fixations.

A post-hoc exploratory analysis using student t-tests (uncorrected for multiple comparisons) shows that the only thresholds that provide a significant difference to the "lowest" filter (threshold = 5) are thresholds 25 and 30. By contrast, post-hoc analyses of the True Positives scores show that the thresholds that are significantly different from threshold 5 are thresholds 9 and upwards.

Together, these results suggest that filters up to threshold 12-15 can produce the highest possible True Positives plus True Negatives, and lowest possible levels of False Positives.

Finally, figure 20 visualizes the normalized proportion of True Positives, True Negatives, False Positives and False Negatives as the velocity threshold and the parameters of discarding and merging are changed.

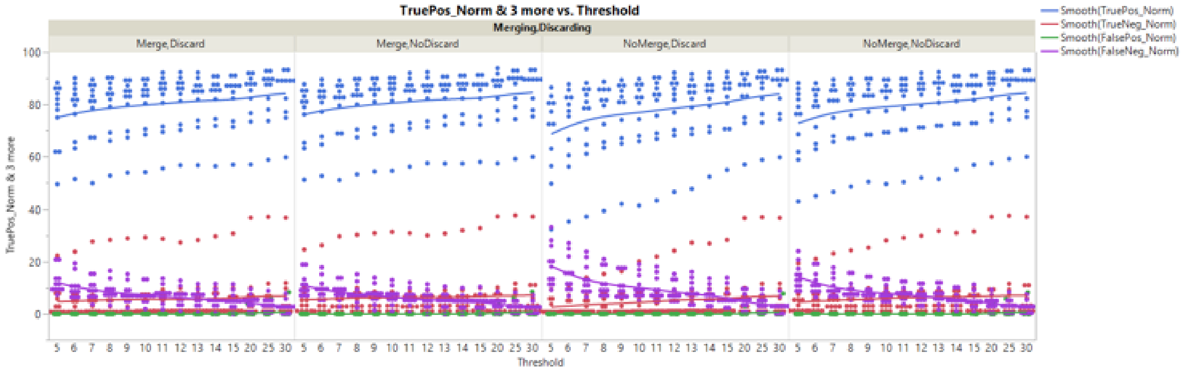

Figure 20: True Positives, True Negatives, False Positives and False Positives combined

1.5 Conclusion

To determine the parameters of the gaze filters to be modified, we ran a pilot test with 5 different viewing conditions where the viewers' selection of gaze was strictly controlled. Our analyses of three different viewing conditions first looked at the following of a single target at 3 sec. speed and then at 1 sec. speed. Following this, we ran a smaller test on the data of fixating between two static targets to see if the findings based on dynamic targets also apply to static targets.

Using Tobii Pro Lab interactive software (figure 5), we observed that while the velocity threshold 5 °/sec is overly strict in terms of classifying gaze points as fixations, the threshold 15°/sec is too liberal and likely to lead to inaccurate data. The optimal velocity threshold was assumed to reside somewhere in between these two border conditions tested and these two values were decided to be taken as the two border conditions. Accordingly, we tested all discrete velocity values ranging from 5 to 15 °/sec as fixation filter parameters and considered True Positives (TP) and False Positives (FP) as most crucial when determining the optimal fixation filter parameters. Also the parameters of discarding short fixations and merging adjacent fixations were included in the analysis.

Taken together, the results for the three different viewing conditions revealed that thresholds below 10°/sec lead to a significant drop in True Positives, whereas velocity thresholds above 11°/sec. introduce an increasing number of False Positives to the data. False Negatives drop significantly from threshold 5°/sec and level off around threshold 10°/sec, and the levels of True Negatives are largely unaffected by threshold levels. For faster scrolling speeds, thresholds above 15°/sec led to better discriminatory values, but as we expect 1-second scroll speeds to be less representative of the data sets for most studies, we believe that the weight of the decision for the optimal threshold should be based on slower scroll speeds and for fixation between two (or more) static targets.

Based on these considerations, it was decided to proceed with the focus on velocity threshold values ranging from 5 to 15 °/sec as fixation filter parameters and run additional studies of larger scale. The pilot study confirmed that the parameters of merging adjacent fixations and discarding short fixations also influence the accuracy of the data output, and therefore need to be included as variables when optimizing the fixation filter parameters for viewing small screens.
